# Photocatalytic Activity of Cellulose Acetate Nanoceria/Pt Hybrid Mats Driven by Visible Light Irradiation

**DOI:** 10.3390/polym13060912

**Published:** 2021-03-16

**Authors:** Federica Costantino, Emanuele Cavaliere, Luca Gavioli, Riccardo Carzino, Luca Leoncino, Rosaria Brescia, Athanassia Athanassiou, Despina Fragouli

**Affiliations:** 1 Smart Materials Group, Istituto Italiano di Tecnologia (IIT), Via Morego 30, 16163 Genova, Italy; federica.costantino@iit.it (F.C.); riccardo.carzino@iit.it (R.C.); athanassia.athanassiou@iit.it (A.A.); 2 Interdisciplinary Laboratories for Advanced Materials Physics (i-LAMP) and Dipartimento di Matematica e Fisica, Università Cattolica del Sacro Cuore, Via Musei 41, 25121 Brescia, Italy; emanuele.cavaliere@unicatt.it (E.C.); luca.gavioli@unicatt.it (L.G.); 3 Electron Microscopy Facility, Istituto Italiano di Tecnologia (IIT), Via Morego 30, 16163 Genova, Italy; luca.leoncino@iit.it (L.L.); rosaria.brescia@iit.it (R.B.)

**Keywords:** ceria nanoparticles, platinum nanoclusters, fiber mats, photodegradation, ROS detection

## Abstract

A photocatalytic system for the degradation of aqueous organic pollutants under visible light irradiation is obtained by an innovative approach based on ceria/platinum (Pt) hybrid nanoclusters on cellulose acetate fibrous membranes. The catalytic materials are fabricated by supersonic beam deposition of Pt nanoclusters directly on the surface of electrospun cellulose acetate fibrous mats, pre-loaded with a cerium salt precursor that is transformed into ceria nanoparticles directly in the solid mats by a simple thermal treatment. The presence of Pt enhances the oxygen vacancies on the surface of the formed ceria nanoparticles and reduces their band gap, resulting in a significant improvement of the photocatalytic performance of the composite mats under visible light irradiation. Upon the appropriate pretreatment and visible light irradiation, we prove that the most efficient mats, with both ceria nanoparticles and Pt nanoclusters, present a degradation efficiency of methylene blue of 70% and a photodegradation rate improved by about five times compared to the ceria loaded samples, without Pt. The present results bring a significant improvement of the photocatalytic performance of polymeric nanocomposite fibrous systems under visible light irradiation, for efficient wastewater treatment applications.

## 1. Introduction

In recent years, considerable research effort has been devoted to the realization of efficient, economical, and green methods for water treatment [1], and heterogeneous photocatalytic oxidation is among the most promising ones [1,2]. This process is widely used for mineralizing many harmful organic species in the presence of materials with photocatalytic properties. Semiconductor nanoparticles (NPs) such as TiO_2_, ZnO, SnO_2_, SiO_2_, etc., show excellent photocatalytic activity due to the combination of large surface area and thermal, electrical, optical, and mechanical properties [1]. However, although most of them are cost effective, with a scalable production, their major drawback is their wide band-gap (E_g_ > 3.2 eV), which limits their photo-response to the ultraviolet (UV) region of the solar spectrum only [3], i.e., just ~5% of the whole solar energy. A possible solution to improve the efficiency of semiconductor NPs is to lower their E_g_ by coupling with metallic NPs, or doping them with transition or noble metals [4,5,6].

Among the diverse semiconductor NPs, ceria (CeO_2_) (CeNPs) are very promising for heterogeneous photocatalytic applications, due to their unique properties such as nontoxicity, chemical stability, low cost, strong oxidizing power, and high electron transfer capability [7]. Ceria excels in forming oxygen vacancies due to the ability to alternate between the Ce^4+^ and Ce^3+^ oxidation states, and to drastically adjust its electronic configuration, and therefore the bandgap, according to its adjacent environment [8]. To form nanomaterials with enhanced photocatalytic performance under visible light irradiation, ceria is combined with various materials such as divalent and trivalent metal oxides, carbon based materials, and metallic NPs [9,10,11]. In particular, platinum (Pt) nanomaterials possess an atomically ordered crystal structure and high catalytic activity [9,12], and therefore the CeO_2_/Pt heterostructures exhibit strong metal-semiconductor interaction effects with a potential to enhance the catalytic activity for reactions involving rapid electron transfer [12]. Such properties improve the efficiency of many reactions such as the CO oxidation, the water-shift reaction, the methanol steam reforming, and the selective oxidation and reduction reactions in the organic liquid phase [2,6]. However, even if CeO_2_/Pt heterostructure NPs have been employed for many applications, their photocatalytic performance for water treatment has been rarely studied [13].

Although NPs possess large surface areas, there are several limitations for their application in water remediation such as the difficulty to manipulate, transport, and recover them from the treated liquids. Furthermore, the NPs aggregation in liquids negatively affects their catalytic activity, due to the significant reduction of their total surface area. A way to overcome these limitations is the immobilization of the NPs on solid supports. Polymers are good host materials for such scope, as they confer flexibility and ease of use to the final material, while supports with high surface area can be formed by electrospinning [14]. However, in most cases, the fabrication processes of the fibrous nanocomposite mats require multi-step procedures comprising the preparation of NPs and the subsequent dispersion in the polymeric solution before the electrospinning process [10]. This may result in mats with limited photocatalytic activity, as the formation of agglomerates and a limited quantity of exposed NPs on the mats’ surface is expected. Alternatively, for the fabrication of fibrous mats for photocatalytic applications, the polymeric matrix has often a “sacrificial role”, and it is eliminated during the conversion of the precursor salt in metal oxide NPs by intense thermal treatment. Following this approach, the final inorganic materials lose the favorable mechanical characteristics offered by the presence of the polymer, compromising their usability [15,16].

An innovative procedure to overcome such limitations is the in situ growth of the NPs within the already formed polymeric mats. The electrospun mats contain a precursor salt that allows the NPs grow upon nucleation when activated by an appropriate thermal treatment or light irradiation ensuring their homogeneous distribution on the surface and inside the fibers [17,18,19]. Following this approach, electrospun fibrous membranes have been used as hosts for the thermal conversion of semiconductor and metallic precursor salts, forming photocatalysts directly immobilized on a structurally stable functional solid network for the photocatalytic degradation of organic dyes [17,18,19]. It is worth noting that depending on the choice of the polymer host, low cost photocatalysts, recyclable or biodegradable, can be formed.

In this work, we report an innovative approach to obtaining a novel photocatalytic system, exploiting Pt nanoclusters (PtNCs) deposition onto a hybrid cellulose acetate (CA) fibrous membrane and subsequent in situ formation of ceria NPs. CA is chosen as the host fibrous matrix due to its bio-based origin, cost effectiveness, and easy manageability. PtNCs are deposited by supersonic cluster beam deposition (SCBD) [20,21,22] on the surface of the electrospun CA mats pre-loaded with a cerium (IV) salt precursor. The post-deposition thermal treatment activates the conversion of the precursor into ceria (CeO_2_) NPs (CeNPs) directly within the fibrous polymeric matrix. We investigate the effect of different PtNC amounts on the CeNPs growth and on the properties of the hybrid mat, as well as on the membranes’ photocatalytic performance under visible light irradiation. We evaluate the effect of the adsorbed methylene blue (MB) on the photocatalytic performance of the mats, and we demonstrate that the presence of PtNCs significantly improves the photocatalytic activity of the CeNPs loaded mats, reaching an overall degradation efficiency of 70%. This is attributed to the CeNPs band gap reduction from 3.3 to 2.4 eV due to the PtNCs. The study of the photodegradation mechanism demonstrates the formation of holes and hydroxyl radicals (^•^OH) as prominent species during the dye degradation. The results demonstrate that, with this versatile method, it is possible to fabricate flexible and easily handled polymeric nanocomposite fibrous mats for visible light-induced photocatalytic applications.

## 2. Materials and Methods

### 2.1. Materials

Cellulose acetate (CA, average MW~40.000 Da), acetone (>99.5%, GC grade), *N,N*-Dimethylformamide (DMF, Cromasolv Plus), isopropyl alcohol (ISO, C_3_H_8_O, <100%), formic acid (FA, CH_2_O_2_, >100%, ACS reagent), fluorescein salt (FL, C_20_H_12_O_5_, >100%), thymoquinone (TQN, C_10_H_12_O_2_, <100%) and Methylene Blue (MB, C_16_H_18_ClN_3_S, <100%) were purchased from Sigma Aldrich (Milano, Italy). Ammonium cerium (IV) nitrate (CePrec, H_8_CeN_8_O_18_, 99.99%), also purchased from Sigma-Aldrich, was chosen as a CeNPs precursor, because it can be combined with polymers and with a mild thermal treatment can induce the formation of CeNPs directly in the solid composites [17]. Furthermore, the specific CePrec has a low toxicity and air stability, is inexpensive and reasonably soluble in many organic media, and can be easily handled [23]. All chemicals were used as received without further purification.

### 2.2. Photocatalytic Mats Preparation

Typically, for the electrospinning process, 1.00 g of CA polymer was dissolved in 10 mL of an Acetone/DMF (volume ratio 7:3) mixture by stirring at 50 °C and 400 rpm (ARE Velp Scientifica, Usmate, Italy) until a clear solution was obtained [18]. Subsequently, 0.107 g of CePrec were dissolved in 5 mL of the same solvent mixture (Acetone/DMF, volume ratio 7:3) upon stirring at 45 °C and 400 rpm for 30 min. Then, the two solutions were mixed and left under stirring for 24 h to obtain a stable solution. Electrospinning was performed in a closed box using a vertical geometry. The solution was placed in a 10 mL syringe, and a syringe pump (NE-1000, New Era pump system Inc., Farmingdale, NY, USA) was used to control the flow rate, while a blunt 19-gauge needle was used as spinneret. The needle was connected to a positive electrode of a high voltage power supply (Glassman High Voltage Inc, High Bridge, NJ, USA), while the aluminum collector was grounded. The spinning voltage, the nozzle-collector distance, and the injection flow rate were 25 kV, 20 cm, and 0.2 mL/h, respectively. The obtained fiber mats (CA/CePrec, with 10.7 %wt. of CePrec in the CA) were dried under low vacuum for 10 h in order to remove any solvent residue.

The surface of the prepared mats was decorated with PtNCs deposited by SCBD, a gas phase synthesis method producing a beam of nanoclusters from a target rod of the desired material [24,25]. In this work, PtNCs were obtained from a Pt rod (purity 99.99%, Alfa Aesar GmbH, Kandel, Germany), using He at pressure P = 50 bar as a carrier gas. The plasma generated by a 0.9 kV pulsed discharge ablates the Pt rod generating NPs that are extracted through a set of aerodynamic lenses and reach the sample’s surface. The amount of the deposited PtNCs is measured by a quartz microbalance and by AFM (Park NX10, Park Systems, Suwon, Korea) on a silicon substrate co-deposited with the mat substrate, and is expressed as an equivalent layer thickness. The thickness of the PtNCs layers deposited on the mats were measured to be either 1 nm or 5 nm.

To promote the nucleation and growth of CeNPs with or without the presence of PtNCs, the fibrous mats with the CePrec were placed in an oven (Froilabo, Lyon, France) at 150 °C for 48 h in air [17]. Considering a total conversion of the precursor salt in metal oxide, the expected CeO_2_ NPs content is approximately ~8% wt. with respect to the polymer. The fabricated mats will be called, for simplicity, CA/CePrec, CA/CeNPs, CA/CeNPs/Pt1, and CA/CeNPs/Pt5, and correspond to the CA mats loaded with precursor, thermally induced grown CeNPs, and thermally induced grown CeNPs with PtNCs at the two different deposition conditions, respectively (see Supplementary Figure S1).

### 2.3. Characterization of Fibrous Mats

All materials were investigated by a JEOL JSM-6490LA scanning electron microscope (SEM, JEOL, Tokyo, Japan) operated at 10 kV beam voltage, equipped with an energy-dispersive X-ray spectroscopy system (EDS). The samples were coated by a 10 nm-thick carbon layer to improve the electrical conductivity.

The morphology and size distribution of the formed NPs was obtained by means of transmission electron microscopy (TEM). To this purpose, the polymeric fibers were dissolved in acetone/DMF (7:3 *v*/*v*), sonicated, and centrifuged in order to remove the polymer. Then, a small volume of the suspension was drop-casted onto a carbon-coated Cu TEM grid. Bright field TEM (BFTEM) images, selected area electron diffraction (SAED) patterns, and annular dark field-scanning TEM (ADF-STEM) images of NPs were acquired by a JEM-1400Plus TEM (JEOL, Tokyo, Japan), operated at 120 kV. This instrument is equipped with a LaB_6_ thermionic electron source, and the images were recorded with a CCD camera (Gatan Orius 830, 2048 × 2048 active pixels, AMETEK, Berwyn, PA, USA), whereas STEM-EDS analyses were performed by means of an EDS system (Dry SD30GV, silicon-drift detector, 30 mm^2^ effective area, JEOL, Tokyo, Japan). The morphology, composition, and crystal structure of the CeNPs/Pt samples were investigated at high spatial resolution by high resolution TEM (HR-TEM) using a JEM-2200FS TEM (JEOL, Tokyo, Japan), operated at 200 kV, equipped with an image-C_S_-corrector, an in-column image filter (Ω-type), and a XFlash 5060 EDS SDD system (60 mm^2^ effective area, Bruker Nano GmbH, Berlin, Germany). The average particles size, the average fibers diameter, and the size distribution (about 50 particles or fibers per sample) were obtained from SEM and TEM images using ImageJ software (National Institutes of Health, Bethesda, MD, USA) [26,27].

X-ray diffraction (XRD) measurements were performed on a PANalytical Empyrean X-ray diffractometer (Malvern Panalytical BV, Almelo, Netherlands) employing the Cu Kα wavelength (λ = 1.5418 Ǻ). The characterization was performed in air and at room temperature in parallel beam geometry and symmetric reflection mode. The data analysis was carried out using PANalytical Highscore 4.1 software (Malvern Panalytical BV, Almelo, Netherlands).

The Raman spectra of the composite mats were acquired by a Raman LabRam HR800 (Horiba Jobin-Yvon Inc., Montpellier, France) spectrometer equipped with a built-in microscope with objective lenses of 10× (NA 0.25) and 50× (NA 0.75), using the 632.8 nm He-Ne laser excitation. The experimental setup consists of a grating 600 lines·mm^−1^ with a spectral resolution of approximately 1 cm^−1^.

UV-Vis diffuse reflectance (DRS) spectroscopy was performed using Varian Cary 6000i (Agilent, Santa Clara, CA, USA) UV-visible-NIR spectrophotometer in the 200–800 nm range, employing an integrating sphere and calibrated with an MgO mirror.

Emission spectra were measured at a room temperature on the solid samples with Fluoromax ^®^-4 spectrofluorometer (Horiba Jobin-Yvon Inc., Montpellier, France) with an excitation wavelength λ_ex_ = 520 nm for all samples.

X-ray photoelectron spectroscopy (XPS) was performed with a monochromatic X-ray source (Lab2, Specs, Berlin, Germany) at 1253 eV and with a hemispherical energy analyzer (Phoibos, HSA3500, Specs, Berlin, Germany) large area lens mode working at ≈1 × 10^−9^ mbar; 90 eV and 30 eV pass energy were employed for the wide scan and for the high-resolution scan, respectively. A flood gun was used to neutralize the surface charge.

### 2.4. Photocatalytic Tests

The photocatalytic activity of all the prepared mats was studied by monitoring the MB concentration change upon irradiation with visible light irradiation. The MB concentration variation was determined with UV-visible absorption spectroscopy using a Varian Cary 6000i spectrometer (Agilent, Santa Clara, CA, USA), by monitoring the evolution of the MB absorption peak at 664 nm and by correlating the intensity with a calibration curve (see Supplementary Figure S2a,b and related discussion).

For each experiment, 3 mL of the MB dye (5 ppm) aqueous solution was placed in a quartz cuvette containing 0.005 g of the fibrous mat suspended in it. Then, the cuvette was placed under a fluorescent lamp (LT-T8, 15W Colourlux plus, Narva, Berlin, Germany) emitting in the 400 nm to 600 nm wavelength. Two types of degradation experiments were conducted. In the first one, before any irradiation, the mats were maintained in contact with the dye solution overnight while in the second case the mats were left in contact with the dye for 1 h, both under dark conditions.

The degradation efficiency (%) was calculated using the following equation:

(1) $$\mathrm{Degradation}\;\mathrm{efficiency}\;\left(\%\right)=\frac{C_{0}-C}{C_{0}}\times 100\%$$

where *C*_0_ is the concentration of the dye after the dark storage and before any irradiation, and *C* is the concentration of the dye solutions at time *t* during the photocatalytic process.

The rate constant of the photodegradation process (*k*, min^−1^) was calculated by fitting the experimental data with the pseudo-first order kinetics model expressed in Equation (2).

(2) $$\ln\;\frac{C_{0}}{C}=kt$$

Before and after the photocatalytic degradation of the MB dye, the total organic carbon (TOC) was determined by the MembraPure uniTOC analyzer (Hennigsdorf, Germany). Before the analysis, all samples were filtered with a PTFE membrane (0.2 μm pore size), and then 1 mL of each solution was further diluted with 20 mL of milliQ H_2_O. The mineralization efficiency (%) is calculated as follows:

(3) $$\mathrm{mineralization}\;\left(\%\right)=\frac{TOC_{i}-TOC_{f}}{TOC_{i}}\cdot 100$$

where *TOC_i_* (mg·L^−1^) and *TOC_f_* (mg·L^−1^) are the TOC concentrations in solution before and after the photodegradation process.

### 2.5. Reactive Oxygen Species Identification

To determine the ^•^OH formation during the visible light irradiation of the fibrous mats, the fluorescein (FL) sodium salt was used as selective radical quencher [3,28]. In particular, for each type of mat, 0.005 g of the sample was added in 3 mL of an aqueous solution of FL (8 μM) in a 1 cm path length quartz cuvette, and then irradiated with visible light for 270 min, in the same conditions as described in the photocatalytic experiments. The ^•^OH formed by the mats upon irradiation react selectively with the FL, and therefore their concentration was calculated using Equation (4), by monitoring the time-dependent reduction of the emission peak of the FL at 515 nm, using a Fluoromax^®^-4 Spectrofluorimeter (excitation wavelength: λ_ex_ = 303 nm). The variation of the emission peak intensity was attributed to the variation of the FL concentration, which was deduced by a calibration process, which correlates the emission intensity of an unknown concentration to the intensity of known FL concentrations (see Supplementary Figure S2c,d). In particular, the evolution of the FL concentration during the visible light irradiation in the presence of the fibrous mat is attributed to its equimolar reaction with the ^•^OH formed by the mats [29]. Therefore, the ^•^OH concentration ([^•^OH]), is calculated by Equation (4):

(4) [OH•]=Δ[Fl]= ΔI/slope

where Δ*I* is the difference of the initial emission intensity *I*_0_, and the intensity at a specific time *I_t_* at *λ_em_* = 515 nm, while the slope is obtained from the calibration curve (see Supplementary Figure S2b,c and Equation (S2)).

For identifying the ROS (reactive oxygen species) during the photocatalysis [29,30], three quenchers were added in an aqueous solution of 5 ppm MB: 1 mM of a hydroxyl radicals quencher (isopropyl alcohol, Sigma-Aldrich, >99.7%), 1 mM of a superoxide radicals quencher (thymoquinone, Sigma-Aldrich, >98%), and 1 mM of an electron holes quencher (formic acid, Sigma-Aldrich, >99%), respectively. For each case, 3 mL of the prepared solution was left in contact with 0.005 g of the mats for 1 h in dark. Subsequently the irradiation with visible light was conducted following the same process as described in the photocatalytic experiments and the absorption peak intensity of MB at 664 nm before and after 270 min of irradiation was recorded.

## 3. Results and Discussion

### 3.1. Morphological Characterization

**Figure 1:**
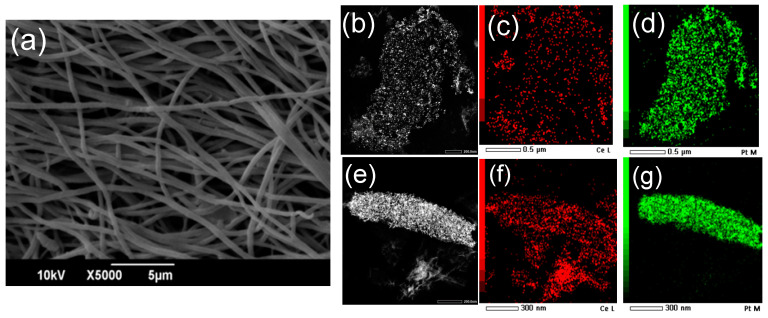
Top view SEM image of (**a**) CA/CeNPs/Pt5. Annular-dark-field STEM images and the corresponding STEM-EDS mapping of the (**b**–**d**) CeNPs/Pt1 and (**e**–**g**) CeNPs/Pt5 (red indicates the Ce and green the Pt element).

All the electrospun mats developed, even after deposition of the PtNCs and thermal treatment, consist of a net of randomly distributed defect-free polymeric fibers in the micron and submicron size range, and irregularly shaped interconnected pores (Figure 1a and Supplementary Figures S3 and S4), proving that the thermal treatment for the CeNPs formation and the deposition of the PtNCs do not alter the mats’ morphology at the microscale. After the thermal treatment, the SEM-EDS mapping shows that the fibers’ composition is homogeneous at the micrometric scale, as well as the Ce and Pt distribution (see Supplementary Figure S5a–n). As determined by SEM-EDS analyses, the Pt/C atomic ratio is more than 10 times higher for the CA/CeNPs/Pt5 mats (0.0034) compared to the CA/CeNPs/Pt1 (0.0003), in line with the SCBD deposition time, while the Pt/Ce atomic ratio is 0.02 and 0.37 for the CA/CeNPs/Pt1 and CA/CeNPs/Pt5, respectively (see Supplementary Table S1).

The coexistence of the Pt and Ce elements at the particles is indicated by the STEM-EDS elemental mapping of the CA/CeNPs/Pt1 and CA/CeNPs/Pt5 samples (Figure 1b–g) after the removal of the CA polymer. In particular, in both cases, features in the form of NPs are evident, and the CeNPs (red) are distributed all over the PtNCs (green), indicating that they grow in close contact with them, as the nanostructures are co-localized, densely packed, and with a uniform distribution.

**Figure 2:**
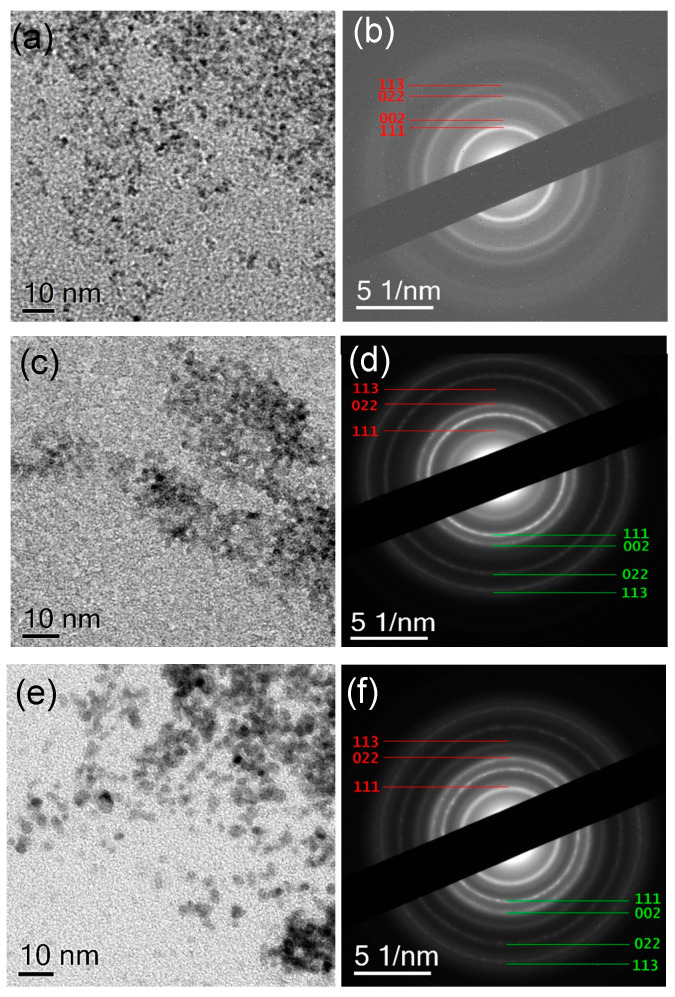
BFTEM images of NPs present in (**a**) CA/CeNPs, (**c**) CA/CeNPs/Pt1, and (**e**) CA/CeNPs/Pt5 samples. To the right the corresponding SAED patterns (**b**,**d**,**f**). The diffraction rings in the patterns are indexed according to the database (ICSD) structures 55284 (CeO_2_) (red) and 243678 (Pt) (green).

In all samples, the CeNPs have spherical shape with mean size ranging between 2–3 nm depending on the sample type (Figure 2). The mean size of the CeNPs formed in the presence of the PtNCs increases slightly with the progression from the Pt-free mats to the Pt rich mats: 2.02 ± 0.61 nm for CA/CeNPs, 2.55 ± 0.66 nm for CA/CeNPs/Pt1, and 3.14 ± 0.85 nm for CA/CeNPs/Pt5 (Supplementary Figure S6 and Table S2). This suggests that the PtNCs presence on the surface of the mats influences the nucleation and growth of CeNPs [17,31]. The SAED patterns of the particles (Figure 2b,d,f) exhibit a sequence of diffraction rings, as expected for randomly oriented nanocrystals. In particular, for all samples, the patterns confirm the presence of crystalline CeNPs (CeO_2_ with a fluorite cubic structure) and the presence of randomly oriented crystalline PtNCs, as clearly evidenced in the CA/CeNPs/Pt1 and CA/CeNPs/Pt5 samples (Figure 2d,f). For the sake of clarity, the profiles obtained by azimuthal integration of SAED patterns are compared with powder X-ray diffraction files obtained from database structures of CeO_2_ and Pt for all samples (see Supplementary Figure S7a–c).

**Figure 3:**
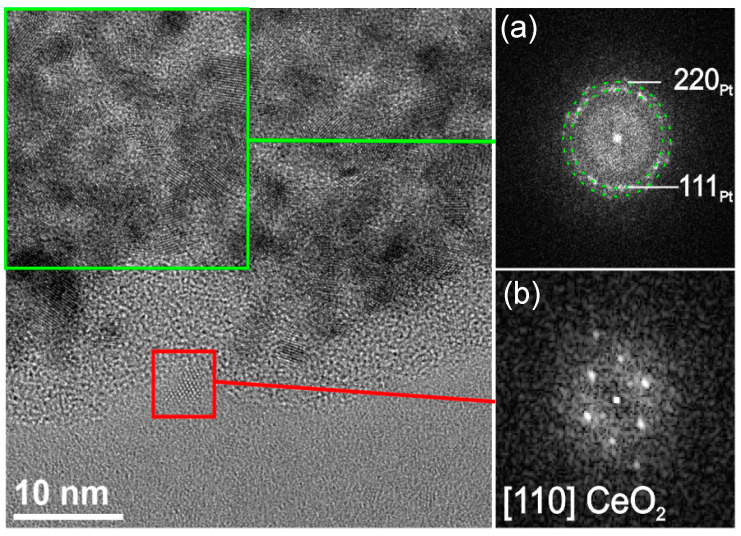
HR-TEM image of the edge region of an aggregate in the sample CA/CeNPs/Pt5 and fast fourier transformation (FFT) analysis of (**a**) a group of PtNCs and (**b**) a CeNP, respectively.

The single-crystal nature of individual Pt and Ce particles is further confirmed by the HR-TEM analysis, and Figure 3 shows the representative example of the NPs present in the CA/CeNPs/Pt5 mats. A closer look to the Figure 3 further demonstrates that the CeNPs grow in contact with the PtNCs, which is also supported by the STEM-EDS analysis (see Supplementary Figure S8), where the CeNPs appear uniformly dispersed and anchored to the PtNCs.

**Figure 4:**
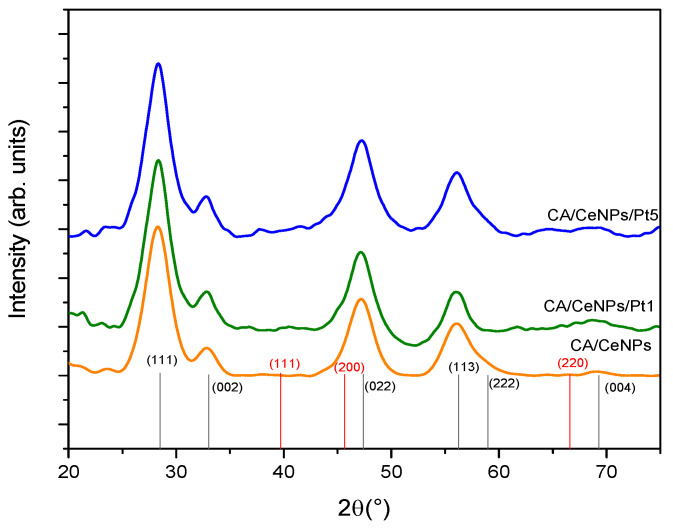
XRD diffractogram of all prepared fiber mats, CA/CeNPs (orange line), CA/CeNPs/Pt1 (green line), and CA/CeNPs/Pt5 (blue line). At the bottom, CeO_2_ patterns ICDD: 96-900-9009 (black) and Pt patters ICDD: 00-004-0802 (red).

The XRD data shown in Figure 4 indicate the prevalence of the CeO_2_ crystalline phase in all the prepared mats. In accordance to SAED profiles (Supplementary Figure S7a), all samples present the main diffraction peaks at 28.5°, 33.1°, 47.5°, 56.3°, and a small peak at 69.4°, which are assigned to the reflections from the (111), (002), (022), (113), and (004) lattice planes of cerium oxide (ICSD: 55284) [32], with no evidence of Ce_2_O_3_ or Ce(OH)_3_ crystalline structures. The Pt crystalline phase is not detected due to the low concentration of the PtNCs as compared to the CeNPs.

**Figure 5:**
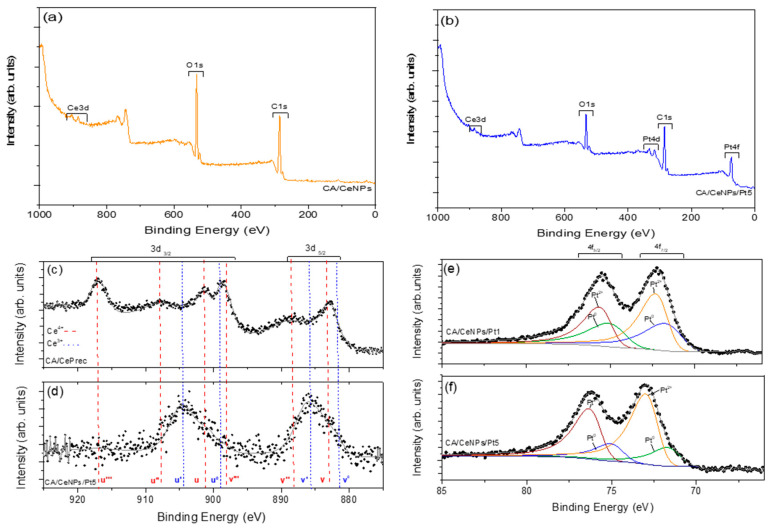
Survey XPS spectrum of (**a**) CA/CeNPs, (**b**) CA/CeNPs/Pt5, and high resolution Ce 3d XPS spectrum of (**c**) CA/CePrec and (**d**) CA/CeNPs/Pt5 (red dashes for Ce4+ and blue dashes for Ce3+). High resolution XPS spectra of platinum (Pt 4f) in the (**e**) CA/CeNPs/Pt1 and (**f**) CA/CeNPs/Pt5.

The surface chemistry of the mats is investigated with XPS. All mats present the Ce3d, O1s, and C1s peaks, with the CA/CeNPs/Pt mats showing also the metallic Pt 4f (Figure 5a,b and Supplementary Figure S9). The atomic concentrations listed in Supplementary Table S3 indicates that C is slightly increased and O is slightly reduced in the presence of Pt. Meanwhile, the Ce % on the surface of the mats is not significantly altered by the presence of the Pt in the surface composition of the CA/CeNPs/Pt mats.

For all the prepared mats, the detailed Ce 3d spectrum exhibits Ce 3d_5/2_ and Ce 3d_3/2_ spin-orbit peaks, indicating the co-existence of Ce^3+^ and Ce^4+^ bonding states [33,34]. When the CePrec is introduced in the CA polymer and the CA/CePrec mats are formed, the composition of the precursor’s Ce oxidation states (Ce^4+^ and Ce^3+^ 70% and 30%, respectively, as deduced from the spectra deconvolution) does not vary (Supplementary Figure S10a and Figure 5c). However, after the thermal treatment the profile of the peaks changes significantly (Figure 5d), indicating that the Ce^3+^ state prevails significantly over the Ce^4+^, and the Ce 3d spectra are quite similar for all the treated samples (see Supplementary Figure S10b,c).

The deconvolution of the O1s spectra of the bare CePrec and CA/CePrec reveals the presence of two distinct peaks. The one at 530.08 eV can be attributed to the Ce^4+^–O lattice, whereas the one at 532.66 eV can be assigned to both the Ce^3+^–O lattice and to the overall oxygen groups in the CA matrix (O=C, O–C, O–H) (see Supplementary Figure S11a–c). In all the other cases, after thermal treatment, the signal attributed to the Ce^4+^–O lattice disappears, and the O1s peak located in the 532.54–532.70 eV range (depending on the sample, Supplementary Figure S11d–f) can be attributed to the overall oxygen groups of the cellulosic membrane [35,36], as well as to the oxygen vacancies possibly due to the prevalent presence of Ce^3+^ on the surface of the mats. Concerning the CA/CeNPs/Pt mats, the Pt 4f spectra (Figure 5e,f) present the typical binding energies of Pt species [37] in the Pt 4f region due to the presence of multiple oxidation states. In particular, the signals of Pt 4f_5/2_ and 4f_7/2_ were deconvoluted into two sets of spin-orbit doublets, indicating the presence of Pt in two forms, the Pt^0^ and Pt^2+^ on the surface of the mats, the latter being present as PtO or Pt(OH)_2_ [30]. Therefore, considering that the Pt deposition on the mats was performed using a fully metallic (Pt^0^) rod in vacuum (for the sake of completeness, the XPS of the Pt rod is reported in Supplementary Figure S12), the subsequent thermally induced growth of the CeNPs in air may cause the modification of its oxidation state [30,31]. In fact, it has been proved that the oxidation state of Pt can be modified by thermal treatment in air, as also by the presence of Ce^3+^/Ce^4+^ and related species [38,39].

**Figure 6:**
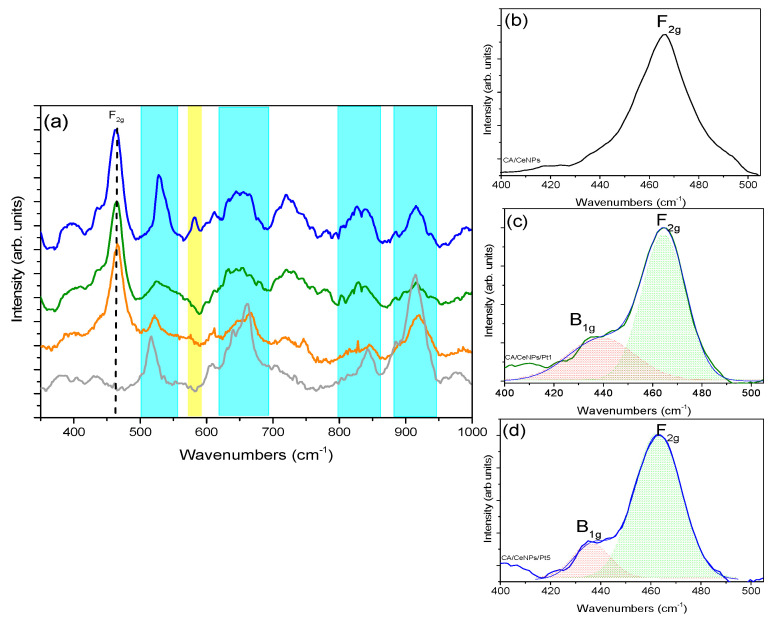
(**a**) Raman spectra of all fibrous mats, CA/CeNPs (orange line), CA/CeNPs/Pt1 (green line), CA/CeNPs/Pt5 (blue line), and CA (therm. treated) matrix (grey line). The blue shadowed peaks correspond to the CA signals and the yellow to the vibrational mode of CeNPs at 580 cm^−^^1^. (**b**) Deconvoluted Raman spectra of CA/CeNPs (**c**), CA/CeNPs/Pt1, and (**d**) CA/CeNPs/Pt5 in the region 400–500 cm^−^^1^ show the F_2g_ peak characteristic of CeO_2_ and the B_2g_ signal referred to PtNCs.

The presence of the trivalent Ce^3+^ ions on the surface of the CA/CeNPs, CA/CeNPs/Pt1, and CA/CeNPs/Pt5 mats, is strictly related to the number of oxygen vacancies and the presence of Ce_2_O_3_ or Ce(OH)_3_ [36,40]. To have an insight on this, the micro-Raman spectra of all prepared mats were acquired, and reported in Figure 6a. In all cases, the signals of the CA polymer matrix appear in the region from 300 to 550 cm^−1^, and are assigned to the C–C–C, O–C–O, and C–C–O deformations [41,42], while the peaks at 918, 834, 659, and 350 cm^−1^ are attributed to the vibrations of the C–H, O–H, C–OH, and C–O bonds, respectively [41]. In comparison to the CA polymer before any treatment, it should be mentioned that some peaks become sharper and more evident after the thermal treatment, due to the formation of crystal domains in the structure of the amorphous CA (Supplementary Figure S13 and Table S4) [43].

Apart from the presence of the characteristic peaks of the CA matrix, the Raman spectra clearly demonstrate the presence of the F_2g_ mode, originating from the symmetric stretching of the octahedral CeO_8_ of the CeO_2_ cubic fluorite structure at about 466.0 cm^−1^ [44,45]. On the basis of the theoretical work performed by Xu et al., the F_2g_ mode is attributed to the breathing vibration of the wagging O atom between two Ce^4+^ ions, and the peak position varies with the length of the Ce–O bonds due to the formation of oxygen defects or the doping metal ions [46]. In the present mats, a slight red shift of the peak is observed when the CeNPs grow in the presence of the PtNCs (from 465.7 cm^−1^ for the CA/CeNPs to 464.6 cm^−1^ for the CA/CeNPs/Pt1 and 463.9 cm^−1^ for the CA/CeNPs/Pt5). The red shift observed can be attributed to the conversion of the Ce^4+^ to the Ce^3+^ oxidation state with localized electrons in the 4f state and the formation of oxygen vacancies in the CeNPs lattice structure in the presence of Pt [31]. In fact, the higher number of oxygen defects in the PtNCs loaded mats results in the slight expansion of the CeNPs supercell, due to the larger radius of the Ce^3+^ (1.07 Å) ions compared to that of Ce^4+^ (0.97 Å), and to the slight elongation of the Ce–O bonds.

Concerning the PtNCs loaded mats, the Pt has two Raman active modes the B_1g_ and E_g_ [47]_._ The B_1g_ is located at ~438 cm^−1^ in both cases, very close to the vibrational signal of ceria, and for this a deconvolution method is adopted to prove its presence, as reported in the Figure 6c,d. The E_g_ mode is located at 657 cm^−1^, but the presence of the polymer matrix makes it difficult to detect. The B_1g_ mode can be related to the presence of PtO species in the surface of mats, in agreement with the XPS analysis. Most importantly, the peak appearing at ~580 cm^−1^ in the CA/CeNPs/Pt5 sample can be assigned to the bridging Pt−O−Ce vibration, i.e., to the interaction between PtNCs and CeNPs [32,38,48,49], strongly supporting the hypothesis that the CeNPs grow in close contact to the PtNCs.

**Figure 7:**
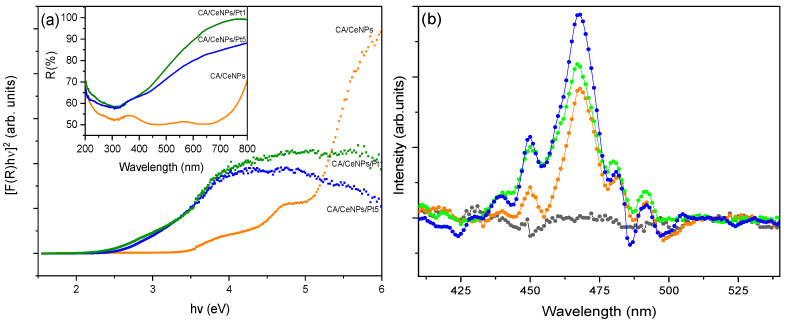
(**a**) Reflectance spectra (inset) and Kubelka–Munk approximation of fibrous mats: CA/CeNPs (orange), CA/CeNPs/Pt1 (green), and CA/CeNPs/Pt5 (blue). (**b**) Fluorescence emission spectra of all prepared fibrous mats: pristine CA (black), CA/CeNPs (orange), CA/CeNPs/Pt1 (green), and CA/CeNPs/Pt5 (blue) (excitation wavelength λ_exc_ = 350 nm).

The evaluation of the E_g_ was carried out by UV-Vis DRS. As shown in the inset of Figure 7a, the R (%) spectrum of the CA/CeNPs presents a peak at 364 nm, which is related to the charge transfer from the 2p level of O to the 4f level of Ce and to the electronic transition 4f→5d in Ce^3+^, while it is not detected when CeNPs grow in the presence of the PtNCs, because the presence of the noble metal causes the enhancement of the absorption in the visible range as shown in the inset of Figure 7a [15,33]. In order to evaluate the E_g_ of all the fiber mats, the Kubelka–Munk approximation was used (details presented in the Supplementary Materials) [50]. Two energy values were derived, which could be attributed to the E_g_ (3.3 eV) and to a possible “mid-gap” (4 eV), due to the energy states generated by defects of the ceria within the gap, mainly by oxygen vacancies that exist in the CeNPs and that could be formed by the CeO_2_ to Ce_2_O_3_ reduction reaction [32]. On the other hand, in the presence of Pt, the E_g_ decreases from 3.3 eV to 2.6 eV and 2.4 eV for CA/CeNPs/Pt1 and CA/CeNPs/Pt5, respectively, denoting that the absorption window of the mats is red-shifted, thus allowing photocatalysis to be activated by visible light.

The modification of the E_g_ and the increase of oxygen vacancies are due to the modification of the valance band in the nanostructure by the coexistence of CeNPs and PtNCs on the surface of the mats [17]. In particular, the decrease of the E_g_ can be attributed to the presence of Ce^3+^ at the grain boundaries with PtNCs, which were able to modify the electronic structure in the oxidation states of CeNPs. The E_g_ decreases with the increase of Ce^3+^ and Pt concentration and by the presence of oxygen vacancies on the surface of the membrane, which are able to form an interface with some localized gap states. On the basis of computational studies [51,52], CeNPs in contact with Pt clusters have modified electronic density. In particular, the Pt facilitates the formation of oxygen vacancies at the metal particles surface, and this implies the creation of centers with propensity for reduction and, at the same time, the formation of holes in the boundaries of cerium, which are good centers for the oxidation process.

To get an insight to the recombination of photogenerated carriers with oxygen vacancies and defects by the coexistence of Ce^3+^/Ce^4+^ species, photoluminescence studies were performed for all the prepared fibrous mats (Figure 7b). The emission peaks between 450 nm and 500 nm are mostly associated with oxygen vacancies, such as F^+^ (oxygen vacancies with one trapped electron), F^2+^ (oxygen vacancies without any electron), and F (oxygen vacancies with one electron). In particular, in the fluorite CeO_2_ structure, the oxygen atoms are not closely packed, so they could form many oxygen vacancies, while maintaining the basic fluorite structure [30]. The CA/CeNPs loaded with PtNCs showed a higher emission intensity with respect to the bare CA/CeNPs, which leads to an enhancement of the optical properties [53]. Therefore, the high intensity can be related to high content of oxygen vacancies and an increase of Ce^3+^ and other defects. In particular, the higher intensity observed in the case of CA/CeNPs/Pt5 than CA/CeNPs/Pt1 suggests a higher separation rate of photoinduced charge carriers, higher separation between the photoelectrons and holes, and subsequently more efficient oxidative process [53].

### 3.2. Photocatalytic Degradation Performance under Visible Light Irradiation

When heterogeneous photocatalysts are used for pollutants removal under light irradiation, both adsorption processes on the photocatalyst material and photocatalytic activity are critical for the photodegradation process [54,55]. Therefore, to establish how the contact of the dye with the fiber mats in the dark may influence the photodegradation mechanism, two different pretreatment conditions were adopted before the light irradiation: the mats and MB solutions are left in contact under dark (i) overnight or (ii) for 1 h.

**Figure 8:**
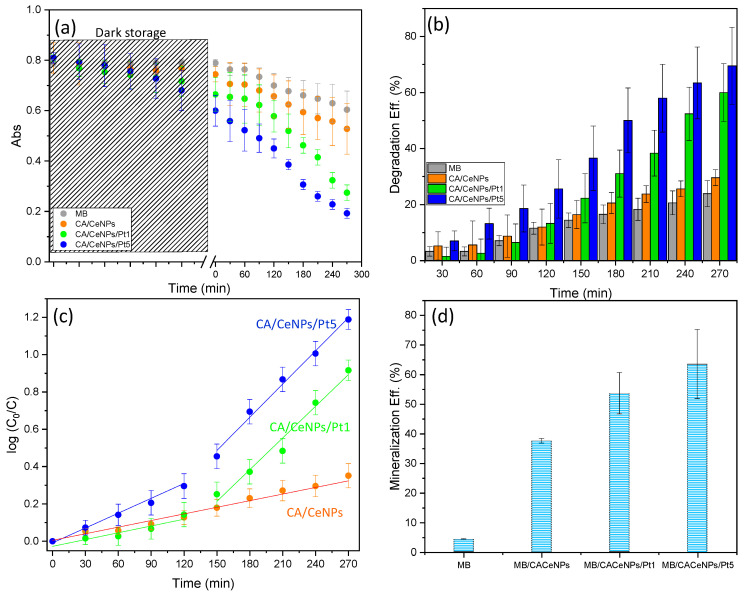
Photodegradation of MB under visible-light irradiation. (**a**) Time dependence of the absorbance at λ = 664 nm (during overnight dark storage, the evolution of the absorbance is monitored at the first 5 h). (**b**) Degradation efficiency (%) vs. time of irradiation. (**c**) Fitting of the pseudo first-order kinetic model to the experimental data. (**d**) MB mineralization efficiency (%) of the MB after the photodegradation process for the different samples. For comparison, the value of the MB solution without any mat and under the same irradiation conditions is also presented.

As can be seen from the Figure 8a (and Supplementary Figure S14), when the mats are left overnight under dark in contact with the MB solution, a decrease of the absorbance is observed in all cases. This can be attributed to the MB physical adsorption of the dye on the surface of the mats by electrostatic interactions with the porous CA network (see Figure S14a,b,f) [18]. Here, the adsorption efficiency for overnight interaction is of c.a. 10% and 8% for the pure CA and for the CA/CeNPs mats, respectively, while in the presence of the PtNCs, it is rather higher (c.a. 18% and 26% for CA/CeNPs/Pt1 and CA/CeNPs/Pt5, respectively), possibly due to the higher surface area and electronegativity of the composite mats, which favor the interaction with the cationic dye [56].

The subsequent visible light irradiation induces significant differences on the degradation performance of the different mats (Figure 8a,b). The CA/CeNPs mat that exhibits low photocatalytic activity ~29%, is notably improved when the CeNPs are grown in the presence of the PtNCs, reaching ~70% for CA/CeNPs/Pt5 and ~60% for CA/CeNPs/Pt1, respectively. This is attributed to the enhanced photocatalytic response of the CeNPs/Pt to the visible light irradiation in combination with the prolonged time of interaction between the dye and the composites, which optimizes the interactions (e.g., adsorption and diffusion) of the organic molecules with the composite mats.

When the interaction of the mats with the dye is adjusted to 1 h under dark, less dye molecules are interacting with the mats, resulting in adsorption efficiencies of ~4% for neat CA fibers mats and CA/CeNPs, ~6% for CA/CeNPs/Pt1, and ~10% for CA/CeNPs/Pt5, respectively. Under these conditions, the overall degradation efficiency is lower compared to the overnight dark storage for all samples (Supplementary Figures S15a–f and S16a–c). Nonetheless, the trend of the degradation performance is quite similar also in this case, with the CA/CeNPs/Pt5 showing the best performance, with an efficiency of ~50%, while for CA/CeNPs/Pt1 and CA/CeNPs it is ~43%, and ~36%, respectively.

The MB degradation kinetics shown in Figure 8c, after the overnight dark contact, reveals a distinct behavior depending on the presence of the Pt NPs. For CA/CeNPs, the ln(C_0_/C) increases linearly with time (with a kinetics rate constant (k) k = 1.3 × 10^−3^ min^−1^). When Pt NPs are present, two different trends emerge. The kinetics rates change from k = 0.7 × 10^−3^ min^−1^ and k = 2.4 × 10^−3^ min^−1^ in the first two hours to k = 4.7 × 10^−3^ min^−1^ and k = 5.9 × 10^−3^ min^−1^ for the CA/CeNPs/Pt1 and the CA/CeNPs/Pt5, respectively (further details in Supplementary Table S5). This can be attributed to the fact that, in the first minutes, the photocatalytic process is mainly focused on the bleaching of the MB adsorbed on the mats, and therefore the MB concentration in the liquid is changing slowly. When the MB is photodegraded, the photocatalytic process accelerates, and the MB in solution is rapidly destroyed. Overall, the PtNCs are increasing the CeNPs responsivity to visible light irradiation.

The role of the adsorption process is evidenced by the photocatalytic kinetics at the mats stored for 1 h under dark, where the slight decrease of the initial MB concentration indicates lower adsorption. The visible light induced decrease of the MB concentration (Supplementary Figure S16) is linear (Equation (2)) for all cases in the entire time scale, proving that the rate change observed after the overnight storage is indeed due to the different dark storage time. The previous reaction rate trend is maintained also in this case, being faster (2.4 × 10^−3^ min^−1^) for the CA/CeNPs/Pt5 than that for CA/CeNPs/Pt1 and CA/CeNPs (1.9 × 10^−3^ min^−1^ and 1.6 × 10^−3^ min^−1^, respectively, see Supplementary Figure S16c and Table S5). It is worth noting that although in the case of CA/CeNPs the difference in the kinetics rate is not so pronounced, possibly due to its low photo reactivity under visible light, confirmed also by the similar photodegradation efficiency; in the case of the CA/CeNPs/Pt mats, the rate is significantly higher when they are stored overnight (second step rate). This may be attributed to the optimized interaction between the dye and the mats surface due to the prolonged contact, but also due to the lower concentration of the MB molecules in the solution due to the adsorption step, which permits the more efficient interaction of the light with the photocatalyst, as the solution is more transparent.

Overall, it can be concluded that the presence of PtNCs on the surface of the mats improves the visible-light photo reactivity, with a better photocatalytic performance for higher Pt concentration. Finally, the MB mineralization efficiency into CO_2_, H_2_O, ions, and some mineral acids is calculated using Equation (3). Figure 8d shows that MB mineralization efficiencies obtained for the CA/CeNPs/Pt5 mats (~63%), and CA/CeNPs/Pt1 mats (~53%) are significantly higher than the one obtained for CA/CeNPs mats (~37%). A similar trend is observed when the mats are left in dark for 1 h, as reported in Supplementary Figure S16d.

### 3.3. Reactive Oxygen Species (ROS) Identification

The photocatalytic effect is stemming from the redox reactions caused by photoinduced charge carriers (e^−^/h^+^) exploited in the formation on the surface of the photocatalysts of reactive oxygen species (ROS), such as superoxide anion radicals, hydrogen peroxide, singlet oxygen, and ^•^OH. Although ^•^OH is the main actor in the mineralization mechanism of persistent organic pollutants [54], it is also important to consider the formation of oxygen superoxide and singlet oxygen species. Moreover, the different photocatalytic activities strongly depend on the kinetics of formation of each ROS [29,57,58].

We evaluated the ^•^OH formation during the photocatalytic process from the FL fluorescence quenching (see experimental section and Supplementary Figure S17 for more details).

**Figure 9:**
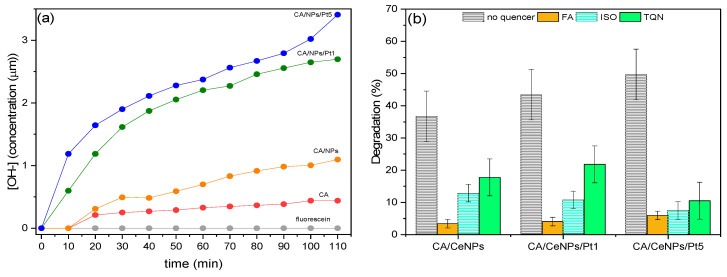
(**a**) Formation of ^•^OH in the MB solution with FL solution (8 μL) without any photocalyst, and by the presence of neat CA mats (red dots), CA/CeNPs (orange dots), CA/CeNPs/Pt1 (blue dots), and CA/CeNPs/Pt5 (green dots). (**b**) Trapping experiment of active species during the MB photodegradation under visible irradiation (λ > 400 nm) of the CA/CeNPs, CA/CeNPsPt1, and CA/CeNPsPt5 mats (0.4 mg·mL^−1^). FA, ISO, and TQNare used to indicate formic acid, isopropyl alcohol, and thymoquinone quenchers, respectively.

As shown in Figure 9a, the time-dependent ^•^OH formation during the visible light irradiation is higher for the CA/CeNPs/Pt5 mats, followed by the CA/CeNPs/Pt1, and then by the CA/CeNPs mats. This confirms the higher responsivity of the Pt loaded mats to the visible light irradiation, in agreement with the photodegradation process trend of MB.

The ^•^OH may be formed by (a) reactions of photogenerated holes or electrons with the water adsorbed on the mat surface or with hydroxyl ions; (b) reactions of protons with superoxide radicals; or (c) photolysis of water, which can be excluded in our case, as it may happen only upon UV light irradiation [28].

To determine the role of the superoxide radicals, the photogenerated holes, and the ^•^OH in the photodegradation process, trapping experiments of the active species during the MB photocatalytic degradation has been performed by adding scavengers in the MB aqueous solution: formic acid (FA) to trap the photogenerated holes, thymoquinone (TQN) for trapping the superoxide radicals, and isopropyl alcohol (ISO) for the ^•^OH. As shown in Figure 9b, the photodegradation efficiency of the mats is significantly decreased in the presence of the scavengers compared to the degradation efficiency of the hybrid mats in the same experimental conditions.

This behavior demonstrates that the dominant factor during the photodegradation process is the holes formation. However, for the CA/CeNPs/Pt5 mats, there is also a notable contribution of the hydroxyl (ISO quencher) and superoxide radicals (TQN quencher). Therefore, the good photocatalytic activity of the CA/CeNPs/Pt5 mats under visible light irradiation can be attributed to the greater number of photogenerated holes, hydroxyl and superoxide radicals, and to the higher rate of their production. This can be attributed to the improved light trapping efficiency given by the combination of CeNPs and PtNCs in the surface of the mats, which causes an electronic modification of the interfaces with a substantial reduction of the bandgap energy and the efficient separation of electron-hole pairs, which are able to trap holes and to produce the ^•^OH on the surface of the CeNPs/PtNCs.

## 4. Conclusions

This study reports an innovative and versatile route for the preparation of polymer-based mats decorated with ceria NPs and platinum nanoclusters for photocatalytic applications. The fabrication is based on the combination of electrospinning and supersonic cluster beam deposition, avoiding the use of hazardous reagents. The thermally induced solid-state reaction in the mats results in the nucleation and growth of CeNPs in contact with the deposited PtNCs. With this process, porous membranes suitable for photocatalytic processes under visible light irradiation are formed, as proved by the study on the photocatalytic degradation of MB, which reached 70% of efficiency. This excellent photocatalytic activity can be attributed to the effective interaction of the MB with the surface of the mats, in combination with the synergistic effect of PtNCs and CeNPs, which are responsible, due to the formation of oxygen vacancies on the surface, for the smaller optical band-gap energy values, combined with the photogenerated ROS species during the photodegradation, especially by the presence of super hydroxyl radicals and holes. Therefore, with this versatile method, it is possible to fabricate flexible and easily handled polymeric nanocomposite fibrous mats with high surface area, for visible light-induced photocatalytic applications.

## Data Availability

The data presented in this study are available on request from the corresponding author.

## Supplementary Materials

The following are available online at https://www.mdpi.com/2073-4360/13/6/912/s1, Figure S1: samples photographs, Figure S2: calibration curves, Figures S3 and S4: morphological and size distribution analysis of the fibers, Figure S5: EDS analysis of the mats, Figure S6: size distribution of the nanoparticles, Figure S7: SAED and XRD analysis of the fibers, Figure S8: HAADF-STEM and EDS mapping, Figures S9–S12: XPS survey spectra and high resolution XPS spectra, Figure S13: Raman analysis of pristine polymer fibers, Figures S14 and S15: MB absorption spectra, Figure S16: photodegradation (%), kinetics behavior and TOC analysis, Figure S17: emission spectra for OH radicals detections. Table S1: atomic percentage (%) of the elements on the mats, Table S2: mean size of the nanoparticles, Table S3: atomic composition of mats derived from XPS, Table S4: Raman bands of the CA, Table S5: Kinetics rate value under visible irradiation, Equations (S1) and (S2): absorption intensity dependence to the concentration for methylene blue and fluorescein, Equations (S3)–(S5): detailed description of the Kubelka-Munk approximation.

## References

[1] Chan S.H.S., Wu T.Y., Juan J.C., Teh C.Y. (2011). Recent Developments of Metal Oxide Semiconductors as Photocatalysts in Advanced Oxidation Processes (AOPs) for Treatment of Dye Waste-Water. J. Chem. Technol. Biotechnol..

[2] Ibhadon A., Fitzpatrick P. (2013). Heterogeneous Photocatalysis: Recent Advances and Applications. Catalysts.

[3] Anastasescu C., Negrila C., Angelescu D.G., Atkinson I., Anastasescu M., Spataru N., Zaharescu M., Balint I. (2018). Particularities of Photocatalysis and Formation of Reactive Oxygen Species on Insulators and Semiconductors: Cases of SiO_2_, TiO_2_ and Their Composite SiO_2_-TiO_2_. Catal. Sci. Technol..

[4] Chatterjee D., Dasgupta S. (2005). Visible Light Induced Photocatalytic Degradation of Organic Pollutants. J. Photochem. Photobiol. C Photochem. Rev..

[5] Kamat P.V. (2002). Photophysical, Photochemical and Photocatalytic Aspects of Metal Nanoparticles. J. Phys. Chem. B.

[6] Wang P., Huang B., Dai Y., Whangbo M.-H. (2012). Plasmonic Photocatalysts: Harvesting Visible Light with Noble Metal Nanoparticles. Phys. Chem. Chem. Phys..

[7] Bellardita M., Fiorenza R., Palmisano L., Scirè S. (2020). Photocatalytic and Photothermocatalytic Applications of Cerium Oxide-Based Materials. Cerium Oxide (CeO₂): Synthesis, Properties and Applications.

[8] Trovarelli A., Llorca J. (2017). Ceria Catalysts at Nanoscale: How Do Crystal Shapes Shape Catalysis?. ACS Catal..

[9] Song S., Wang X., Zhang H. (2015). CeO2-Encapsulated Noble Metal Nanocatalysts: Enhanced Activity and Stability for Catalytic Application. NPG Asia Mater..

[10] Morselli D., Valentini P., Perotto G., Scarpellini A., Pompa P.P., Athanassiou A., Fragouli D. (2017). Thermally-Induced in Situ Growth of ZnO Nanoparticles in Polymeric Fibrous Membranes. Compos. Sci. Technol..

[11] Hoque M.A., Guzman M.I. (2018). Photocatalytic Activity: Experimental Features to Report in Heterogeneous Photocatalysis. Materials.

[12] Jia H., Zhu X.-M., Jiang R., Wang J. (2017). Aerosol-Sprayed Gold/Ceria Photocatalyst with Superior Plasmonic Hot Electron-Enabled Visible-Light Activity. ACS Appl. Mater. Interfaces.

[13] Fandi Z., Ameur N., Brahimi F.T., Bedrane S., Bachir R. (2020). Photocatalytic and Corrosion Inhibitor Performances of CeO2 Nanoparticles Decorated by Noble Metals_ Au, Ag, Pt. J. Environ. Chem. Eng..

[14] Thamer B.M., Aldalbahi A., Moydeen A.M., Rahaman M., El-Newehy M.H. (2021). Modified Electrospun Polymeric Nanofibers and Their Nanocomposites as Nanoadsorbents for Toxic Dye Removal from Contaminated Waters: A Review. Polymers.

[15] Li C., Chen R., Zhang X., Shu S., Xiong J., Zheng Y., Dong W. (2011). Electrospinning of CeO_2_-ZnO Composite Nanofibers and Their Photocatalytic Property. Mater. Lett..

[16] Sharma R., Singh N., Gupta A., Tiwari S., Tiwari S.K., Dhakate S.R. (2014). Electrospun Chitosan–Polyvinyl Alcohol Composite Nanofibers Loaded with Cerium for Efficient Removal of Arsenic from Contaminated Water. J. Mater. Chem. A.

[17] Morselli D., Campagnolo L., Prato M., Papadopoulou E.L., Scarpellini A., Athanassiou A., Fragouli D. (2018). Ceria/Gold Nanoparticles in Situ Synthesized on Polymeric Membranes with Enhanced Photocatalytic and Radical Scavenging Activity. ACS Appl. Nano Mater..

[18] Costantino F., Armirotti A., Carzino R., Gavioli L., Athanassiou A., Fragouli D. (2020). In Situ Formation of SnO_2_ Nanoparticles on Cellulose Acetate Fibrous Membranes for the Photocatalytic Degradation of Organic Dyes. J. Photochem. Photobiol. A Chem..

[19] Campagnolo L., Lauciello S., Athanassiou A., Fragouli D. (2019). Au/ZnO Hybrid Nanostructures on Electrospun Polymeric Mats for Improved Photocatalytic Degradation of Organic Pollutants. Water.

[20] Peli S., Cavaliere E., Benetti G., Gandolfi M., Chiodi M., Cancellieri C., Giannetti C., Ferrini G., Gavioli L., Banfi F. (2016). Mechanical Properties of Ag Nanoparticle Thin Films Synthesized by Supersonic Cluster Beam Deposition. J. Phys. Chem. C.

[21] Fraters B.D., Cavaliere E., Mul G., Gavioli L. (2014). Synthesis of Photocatalytic TiO_2_ Nano-Coatings by Supersonic Cluster Beam Deposition. J. Alloy. Compd..

[22] Benetti G., Cavaliere E., Brescia R., Salassi S., Ferrando R., Vantomme A., Pallecchi L., Pollini S., Boncompagni S., Fortuni B. (2019). Tailored Ag-Cu-Mg Multielemental Nanoparticles for Wide-Spectrum Antibacterial Coating. Nanoscale.

[23] Nair V., Deepthi A. (2007). Cerium(IV) Ammonium NitrateA Versatile Single-Electron Oxidant. Chem. Rev..

[24] Benetti G., Cavaliere E., Banfi F., Gavioli L. (2020). Antimicrobial Nanostructured Coatings: A Gas Phase Deposition and Magnetron Sputtering Perspective. Materials.

[25] Chiodi M., Cheney C.P., Vilmercati P., Cavaliere E., Mannella N., Weitering H.H., Gavioli L. (2012). Enhanced Dopant Solubility and Visible-Light Absorption in Cr-N Codoped TiO_2_ Nanoclusters. J. Phys. Chem. C.

[26] Rueden C.T., Schindelin J., Hiner M.C., DeZonia B.E., Walter A.E., Arena E.T., Eliceiri K.W. (2017). ImageJ2: ImageJ for the next Generation of Scientific Image Data. BMC Bioinform..

[27] Mitchell D.R.G. (2008). DiffTools: Electron Diffraction Software Tools for DigitalMicrograph^TM^. Microsc. Res. Tech..

[28] Serrà A., Zhang Y., Sepúlveda B., Gómez E., Nogués J., Michler J., Philippe L. (2019). Highly Active ZnO-Based Biomimetic Fern-like Microleaves for Photocatalytic Water Decontamination Using Sunlight. Appl. Catal. B Environ..

[29] Nosaka Y., Nosaka A.Y. (2017). Generation and Detection of Reactive Oxygen Species in Photocatalysis. Chem. Rev..

[30] Manwar N.R., Chilkalwar A.A., Nanda K.K., Chaudhary Y.S., Subrt J., Rayalu S.S., Labhsetwar N.K. (2016). Ceria Supported Pt/PtO-Nanostructures: Efficient Photocatalyst for Sacrificial Donor Assisted Hydrogen Generation under Visible-NIR Light Irradiation. ACS Sustain. Chem. Eng..

[31] Daelman N., Capdevila-Cortada M., López N. (2019). Dynamic Charge and Oxidation State of Pt/CeO_2_ Single-Atom Catalysts. Nat. Mater..

[32] Calvache-Muñoz J., Prado F.A., Tirado L., Daza-Gomez L.C., Cuervo-Ochoa G., Calambas H.L., Rodríguez-Páez J.E. (2019). Structural and Optical Properties of CeO_2_ Nanoparticles Synthesized by Modified Polymer Complex Method. J. Inorg. Organomet. Polym. Mater..

[33] Ma R., Jahurul Islam M., Amaranatha Reddy D., Kim T.K. (2016). Transformation of CeO_2_ into a Mixed Phase CeO_2_/Ce_2_O_3_ Nanohybrid by Liquid Phase Pulsed Laser Ablation for Enhanced Photocatalytic Activity through Z-Scheme Pattern. Ceram. Int..

[34] Paparazzo E. (2018). Use and Mis-Use of X-ray Photoemission Spectroscopy Ce3d Spectra of C_2_O_3_ and CeO_2_. J. Phys. Condens. Matter.

[35] Ansari S.A., Khan M.M., Ansari M.O., Kalathil S., Lee J., Cho M.H. (2014). Band Gap Engineering of CeO_2_ Nanostructure Using an Electrochemically Active Biofilm for Visible Light Applications. RSC Adv..

[36] Qi L., Yu Q., Dai Y., Tang C., Liu L., Zhang H., Gao F., Dong L., Chen Y. (2012). Influence of Cerium Precursors on the Structure and Reducibility of Mesoporous CuO-CeO_2_ Catalysts for CO Oxidation. Appl. Catal. B Environ..

[37] Romanchenko A., Likhatski M., Mikhlin Y. (2018). X-ray Photoelectron Spectroscopy (XPS) Study of the Products Formed on Sulfide Minerals Upon the Interaction with Aqueous Platinum (IV) Chloride Complexes. Minerals.

[38] Lee J., Ryou Y., Chan X., Kim T.J., Kim D.H. (2016). How Pt Interacts with CeO_2_ under the Reducing and Oxidizing Environments at Elevated Temperature: The Origin of Improved Thermal Stability of Pt/CeO_2_ Compared to CeO_2_. J. Phys. Chem. C.

[39] Mao M., Lv H., Li Y., Yang Y., Zeng M., Li N., Zhao X. (2016). Metal Support Interaction in Pt Nanoparticles Partially Confined in the Mesopores of Microsized Mesoporous CeO_2_ for Highly Efficient Purification of Volatile Organic Compounds. ACS Catal..

[40] Filtschew A., Hofmann K., Hess C. (2016). Ceria and Its Defect Structure: New Insights from a Combined Spectroscopic Approach. J. Phys. Chem. C.

[41] Agarwal U.P. (2019). Analysis of Cellulose and Lignocellulose Materials by Raman Spectroscopy: A Review of the Current Status. Molecules.

[42] Fischer S., Schenzel K., Fischer K., Diepenbrock W. (2005). Applications of FT Raman Spectroscopy and Micro Spectroscopy Characterizing Cellulose and Cellulosic Biomaterials. Macromol. Symp..

[43] Zhang K., Feldner A., Fischer S. (2011). FT Raman Spectroscopic Investigation of Cellulose Acetate. Cellulose.

[44] Sartoretti E., Novara C., Giorgis F., Piumetti M., Bensaid S., Russo N., Fino D. (2019). In Situ Raman Analyses of the Soot Oxidation Reaction over Nanostructured Ceria-Based Catalysts. Sci. Rep..

[45] Kosacki I., Suzuki T., Anderson H.U., Colomban P. (2002). Raman Scattering and Lattice Defects in Nanocrystalline CeO2 Thin Films. Solid State Ion..

[46] Xu Y., Wang F., Liu X., Liu Y., Luo M., Teng B., Fan M., Liu X. (2019). Resolving a Decade-Long Question of Oxygen Defects in Raman Spectra of Ceria-Based Catalysts at Atomic Level. J. Phys. Chem. C.

[47] McBride J.R., Graham G.W., Peters C.R., Weber W.H. (1991). Growth and Characterization of Reactively Sputtered Thin-film Platinum Oxides. J. Appl. Phys..

[48] Derevyannikova E.A., Kardash T.Y., Stadnichenko A.I., Stonkus O.A., Slavinskaya E.M., Svetlichnyi V.A., Boronin A.I. (2019). Structural Insight into Strong Pt-CeO_2_ Interaction: From Single Pt Atoms to PtO_x_ Clusters. J. Phys. Chem. C.

[49] Lin W., Herzing A.A., Kiely C.J., Wachs I.E. (2008). Probing Metal−Support Interactions under Oxidizing and Reducing Conditions: In Situ Raman and Infrared Spectroscopic and Scanning Transmission Electron Microscopic−X-ray Energy-Dispersive Spectroscopic Investigation of Supported Platinum Catalysts. J. Phys. Chem. C.

[50] López R., Gómez R. (2012). Band-Gap Energy Estimation from Diffuse Reflectance Measurements on Sol–Gel and Commercial TiO2: A Comparative Study. J. Sol. Gel Sci. Technol..

[51] Bruix A., Migani A., Vayssilov G.N., Neyman K.M., Libuda J., Illas F. (2011). Effects of Deposited Pt Particles on the Reducibility of CeO_2_ (111). Phys. Chem. Chem. Phys..

[52] Tao L., Shi Y., Huang Y.-C., Chen R., Zhang Y., Huo J., Zou Y., Yu G., Luo J., Dong C.-L. (2018). Interface Engineering of Pt and CeO_2_ Nanorods with Unique Interaction for Methanol Oxidation. Nano Energy.

[53] Khan M.M., Ansari S.A., Pradhan D., Han D.H., Lee J., Cho M.H. (2014). Defect-Induced Band Gap Narrowed CeO_2_ Nanostructures for Visible Light Activities. Ind. Eng. Chem. Res..

[54] Chiu Y.-H., Chang T.-F.M., Chen C.-Y., Sone M., Hsu Y.-J. (2019). Mechanistic Insights into Photodegradation of Organic Dyes Using Heterostructure Photocatalysts. Catalysts.

[55] Andronic L., Isac L., Cazan C., Enesca A. (2020). Simultaneous Adsorption and Photocatalysis Processes Based on Ternary TiO_2_-Cu_x_S-Fly Ash Hetero-Structures. Appl. Sci..

[56] Ranjith K.S., Satilmis B., Huh Y.S., Han Y.-K., Uyar T. (2020). Highly Selective Surface Adsorption-Induced Efficient Photodegradation of Cationic Dyes on Hierarchical ZnO Nanorod-Decorated Hydrolyzed PIM-1 Nanofibrous Webs. J. Colloid Interface Sci..

[57] Nosaka Y., Nosaka A. (2016). Introduction to Photocatalysis: From Basic Science to Applications.

[58] Ribao P., Corredor J., Rivero M.J., Ortiz I. (2019). Role of Reactive Oxygen Species on the Activity of Noble Metal-Doped TiO_2_ Photocatalysts. J. Hazard. Mater..

