# Supplementary material for: Photocatalytic Activity of Cellulose Acetate Nanoceria/Pt Hybrid Mats Driven by Visible Light Irradiation
Source: Polymers (Basel). 2021 Mar 16;13(6):912. doi: 10.3390/polym13060912 (PMC8002269; doi:10.3390/polym13060912)
Supplement: Supplementary file 1 [file polymers-13-00912-s001.pdf]

polymers

MDPI

Supplementary Materials

Photocatalytic Activity of Cellulose Acetate nanoceria/Pt hybrid mats driven by Visible Light Irradiation

Federica Costantino [1,2], Emanuele Cavaliere [2], Luca Gavioli [2], Riccardo Carzino [1], Luca Leoncino [3], Rosaria Brescia [3], Athanassia Athanassiou [1] and Despina Fragouli [1,*]

[1] Smart Materials Group, Istituto Italiano di Tecnologia (IIT), Via Morego 30, 16163, Genova, Italy
[2] [b]Interdisciplinary Laboratories for Advanced Materials Physics (i-LAMP) and Dipartimento di Matematica e Fisica, Università Cattolica del Sacro Cuore, Via Musei 41, 25121 Brescia, Italy
[3] Electron Microscopy Facility, Istituto Italiano di Tecnologia, Via Morego 30, 16163 Genova, Italy

Table of contents

Figure S1: samples photographs, Figure S2: calibration curves, Figures S3-S4: morphological and size distribution of the fibers, Figure S5: EDS analysis of the mats. Table S1: atomic percentage (%) of element on the mats. Figure S6: size analysis distribution of nanoparticles. Table S2: size of NPs derived from TEM and XRD. Figure S7: SAED and XRD analysis of the fibers, Figure S8: HAADF-STEM and EDS mapping, Table S3: atomic composition of the mats derived from XPS; Figure S8-S12: XPS survey spectra and high resolution XPS spectra. Figure S13: Raman analysis of the pristine polymer fibers; Table S4: Raman bands of CA matrix. Eq S3-S5: detailed description of the Kubelka-Munk approximation, Figure S14-S15: MB absorption spectra; Figure S16: photodegradation (%), kinetic behavior and TOC analysis. Table S5: Kinetic rate constant under visible light irradiation. Figure S17: emission spectra for OH radicals.

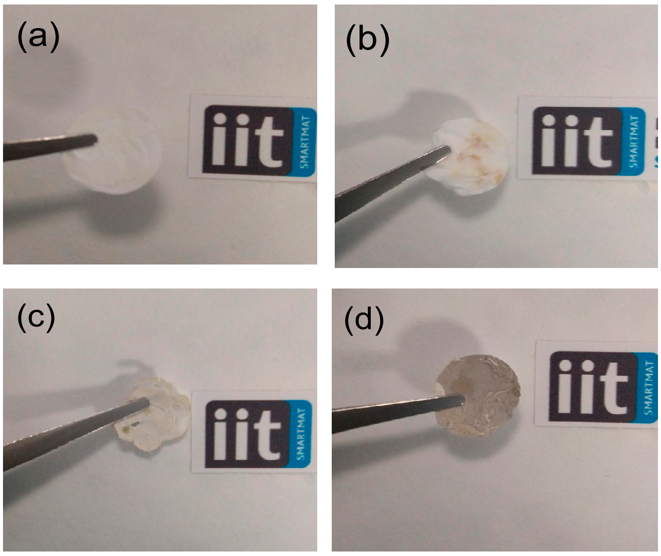

Figure S1. Photographs of all prepared fibrous membrane (a) CA/CePrec, (b) CA/CeNPs, (c) CA/CeNPs/Pt1 and (d) CA/CeNPs/Pt5.

Calibration curve

Initially the absorption spectra of aqueous solutions containing different concentrations of MB (from 0.1 to 5 ppm) and the emission spectra of aqueous solutions containing different concentrations of fluorescein (from 8 um to 0.5 μm) were recorded (Figure S2a,c). The intensity of the representative absorption peak of MB (λ_{abs}=664nm) and of the emission peak of fluorescein (λ_{em}=515nm) for each concentration were then plotted (Figure S1b,d) and the linear fitting in each case was conducted. Therefore, for MB the absorption intensity dependence to the concentration is defined by the equation S1 and for Fluorescein by equation S2.

$y= 0.14+0.014x$ Eq. S1

$y= 3.25\cdot10^{-6}x$ Eq. S2

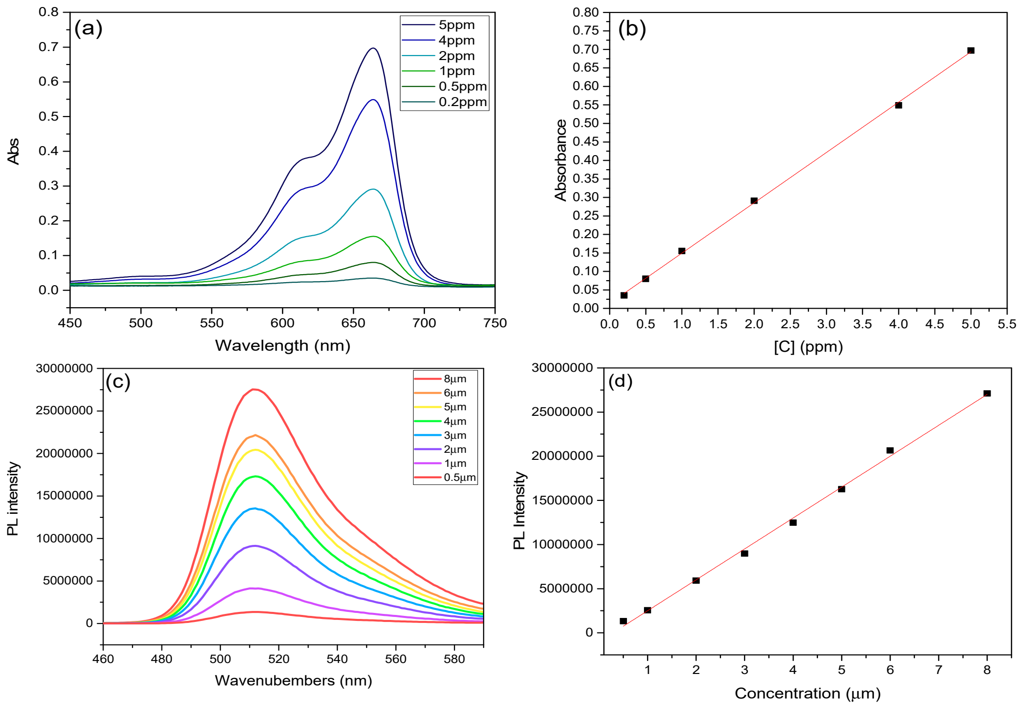

Figure S2. (a) Absorption spectra and (b) calibration curve of MB. (c,d) Emission spectra and related calibration curve of FL respectively.

Morphological and size distribution analysis

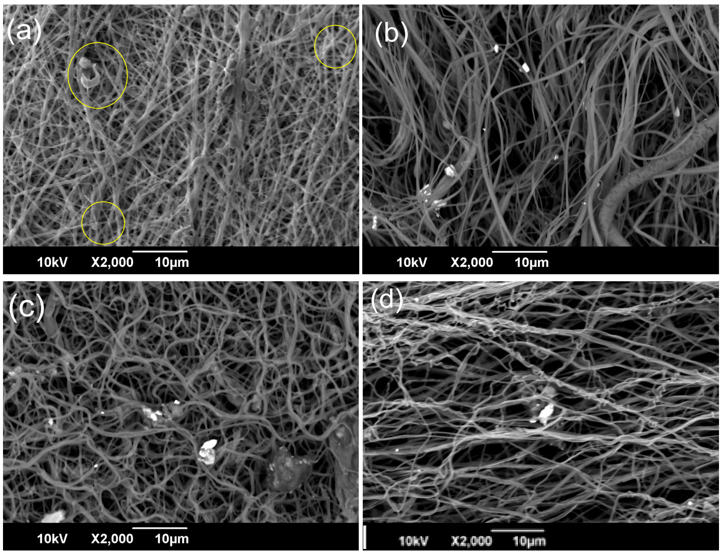

Figure S3.Top view SEM images (back scattered electron signal) of (a) CA/CePrec, (b) CA/CeNPs, (c) CA/CeNPs/Pt1 and (d) CA/CeNPs/Pt5 (scale bar 10 μm).

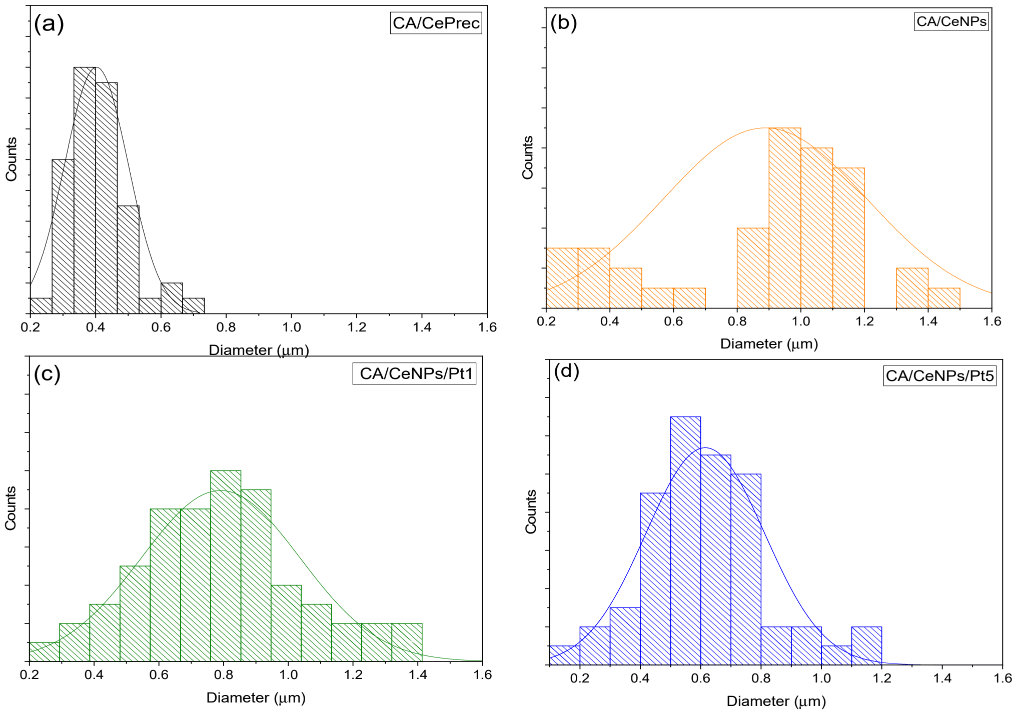

Figure S4. Size distribution of the fibers: (a) CA/CePrec, (b) CA/CeNPs, (c) CA/CeNPs/Pt1 and (d) CA/CeNPs/Pt5.

EDS mapping of the fiber mats

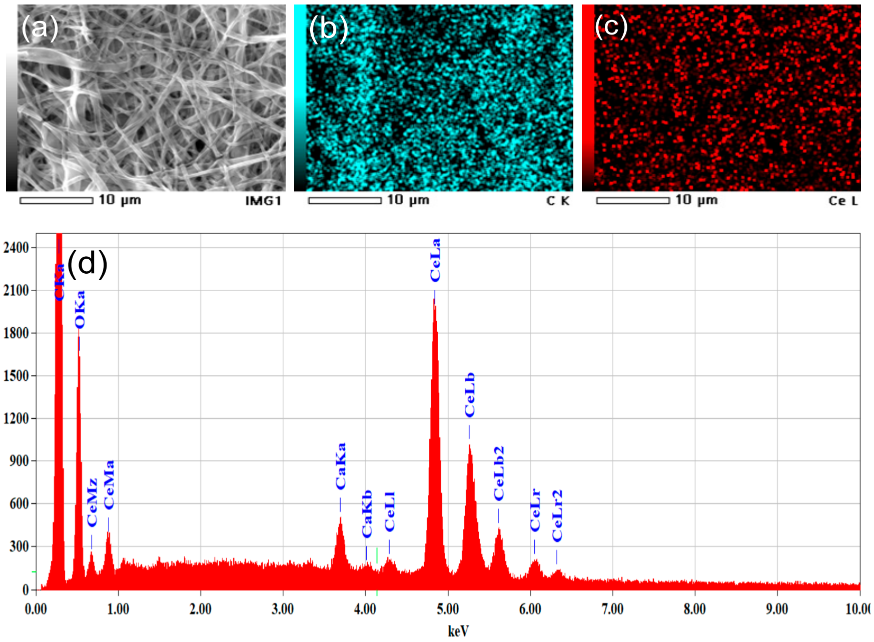

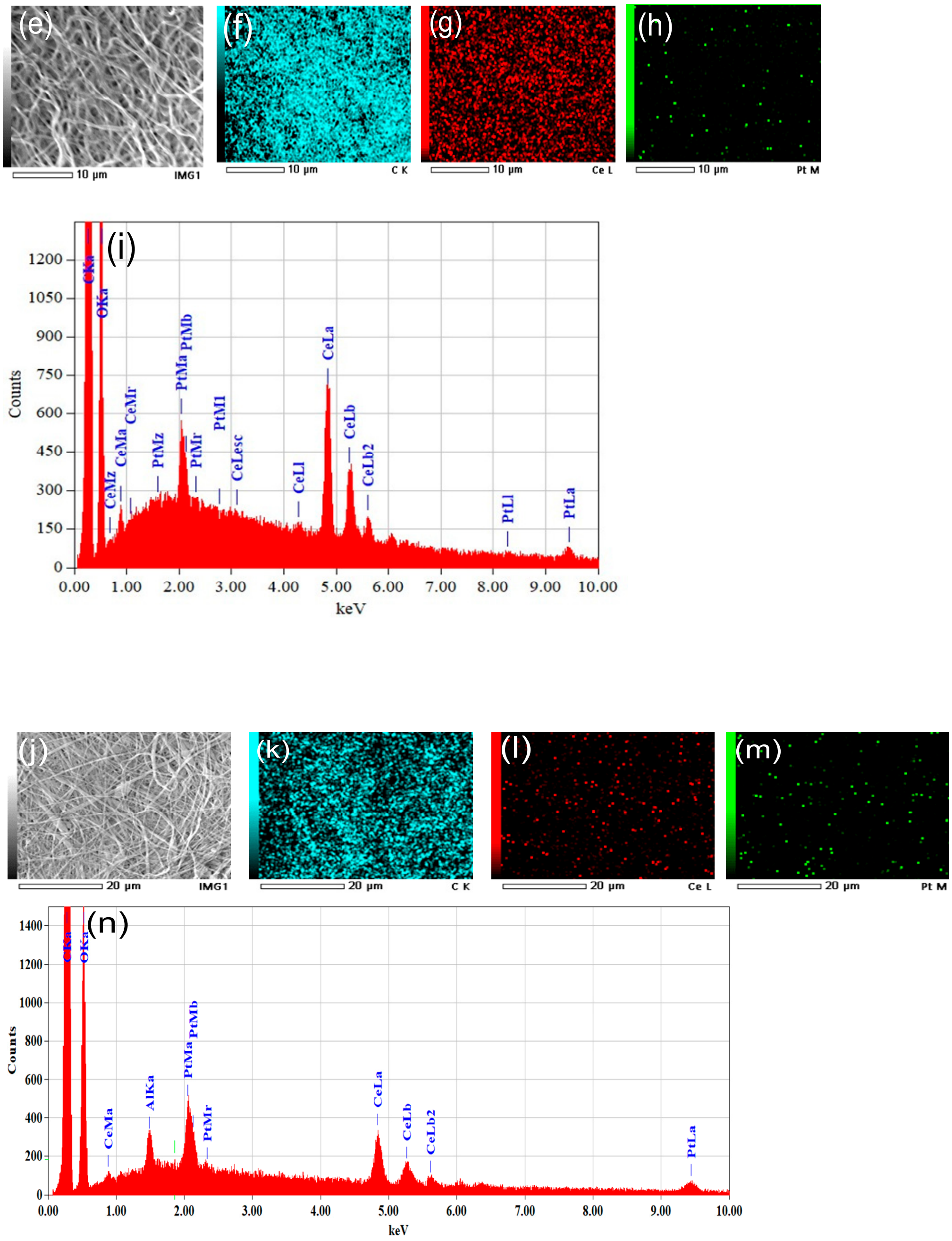

Figure S5. (a) SEM image of CA/CeNPs and corresponding EDS mapping of the mats; elemental mapping for (b) C and (c) Ce. (d) EDS spectrum related to the area shown in image (a). (e) SEM image of CA/CeNPs/Pt1 and related EDS mapping of the mats for (f) C, (g) Ce and (h) Pt, (i) EDS spectrum related to the area shown in image (e). (j) SEM image of CA/CeNPs/Pt5 and related EDS mapping of the mats (k) for C, (l) Ce and (m) Pt, (n) EDS spectrum related to the area shown in image (j).

Table S1. Atomic percentage (%) of the elements on the mats (according to SEM-EDS analysis)

**Samples**	**C (%)**	**O (%)**	**Ce (%)**	**Pt (%)**
CA/CeNPs	75.87	22.62	1.03	-
CA/CeNPs/Pt1	76.70	21.81	1.06	0.02
CA/CeNPs/Pt5	73.13	25.51	0.67	0.25

Morphological analysis and mapping of CeNPs/PtNCs

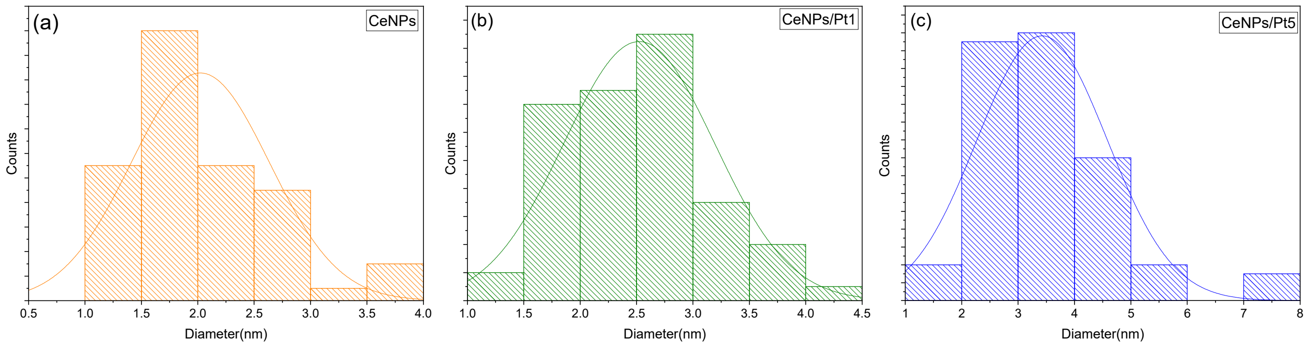

Figure S6. Size distribution of the NPs (a) CeNPs, (b) CeNPs/Pt1, (c) CeNPs/Pt5.

Table S2. Size of NPs calculated from TEM analysis.

**Method**	**TEM**
Samples	Size (nm)
CA/CeNPs	2.0±0.6
CA/CeNPs/Pt1	2.6±0.7
CA/CeNPs/Pt5	3.1±0.8

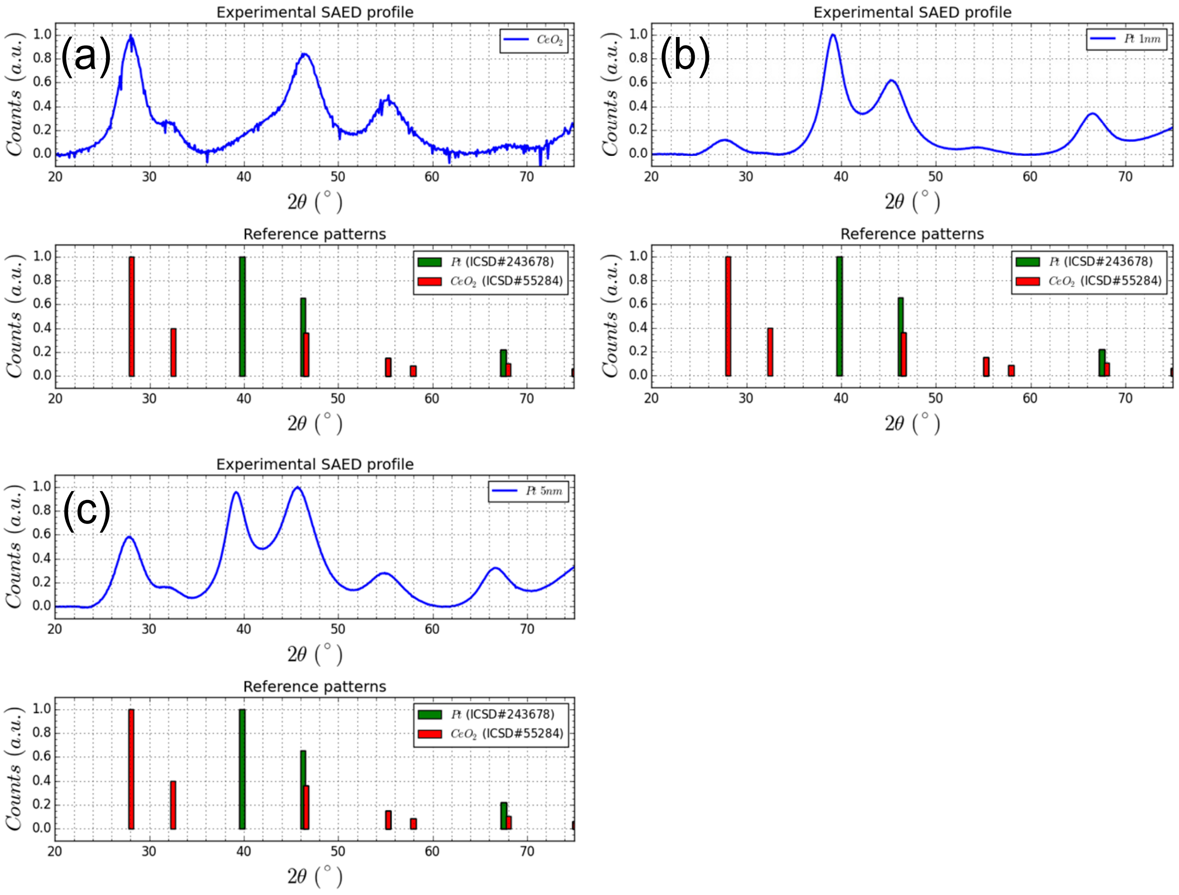

Figure S7. Profiles obtained by azimuthal integration, followed by background subtraction, from SAED patterns of (a) CeNPs, (b) CeNPs/Pt1 and (c) CeNPs/Pt5, proving the presence of crystalline CeNPs and PtNCs in accordance with the references (ICSD:#55284) and (ICSD:#243678) respectively. Processing of SAED patterns (azimuthal integration + background subtraction) was carried out using the PASAD plugin of Gatan Digital Micrograph [1]. The 2θ Bragg value was calculated assuming the kα line of Cu, for ease of comparison with XRD data.

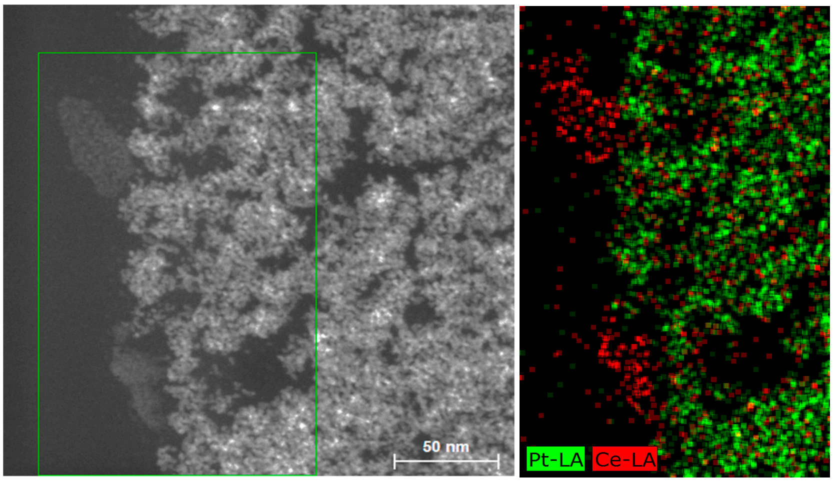

Figure S8. (Left) HAADF-STEM image and (right) corresponding STEM-EDS mapping of the selected area of the CA/CeNPs/Pt5 sample.

XPS analysis

Table S3. Atomic compositions (%) of all prepared fiber mats

Samples	C (%)	O (%)	Ce (%)	Pt (%)
CA	69.78	30.21	/	/
CA/CeNPs	69.95	29.72	0.32	/
CA/CeNPs/Pt1	71.47	23.43	0.24	3
CA/CeNPs/Pt5	76.47	20.21	0.30	4

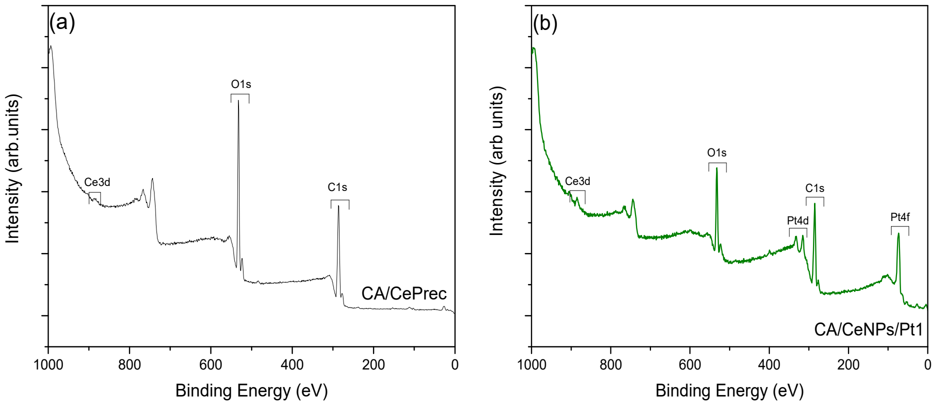

Figure S9. (a) Survey spectra of CA/CePrec, and (b) CA/CeNPs/Pt1.

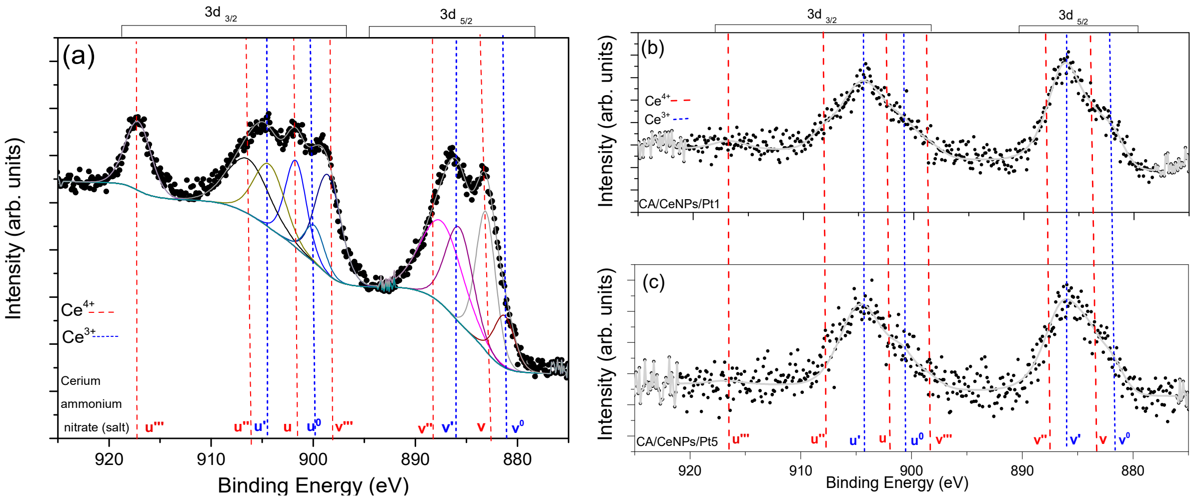

Figure S10. High-resolution spectrum of Ce3d region (Ce of (a) CePrec salt, (b) CA/CeNPs/Pt1 and (c) CA/CeNPs/Pt5 (red dashed line for Ce^{4+} and blue dashed line for Ce^{3+}).

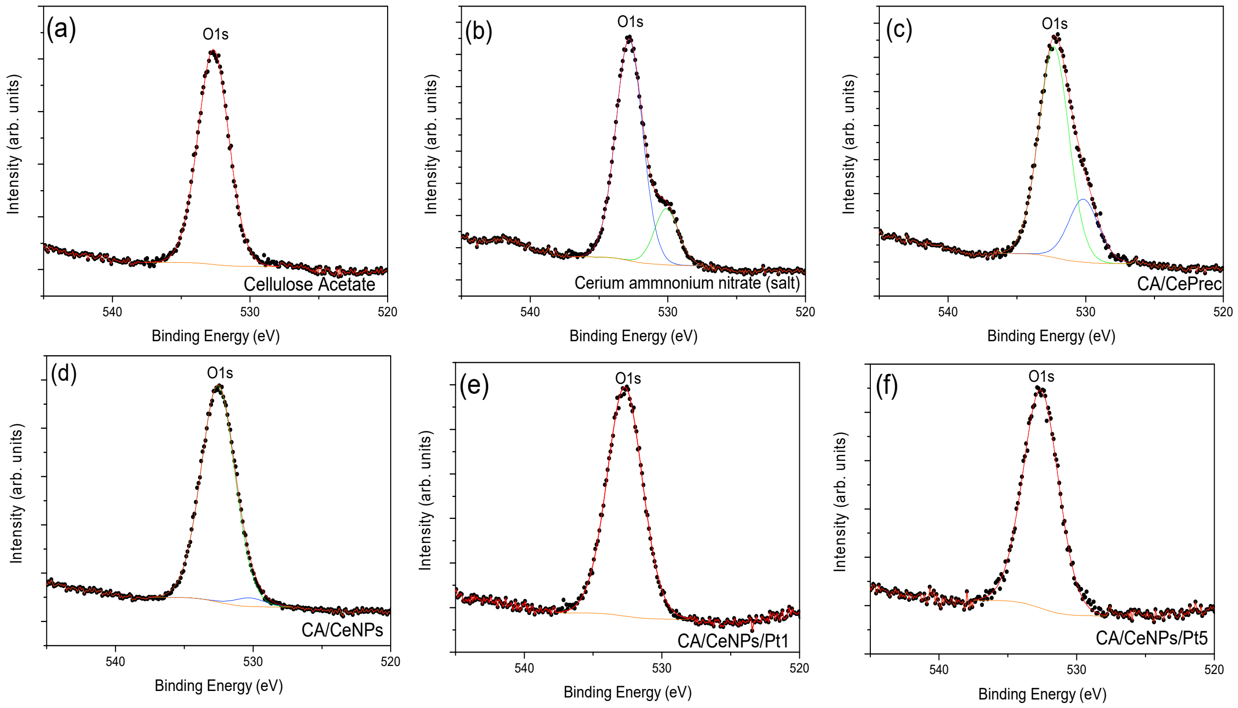

Figure S11. O1s XPS spectra of (a) CePrec (salt), (b) CA/CePrec, (c) CA/CeNPs, (d) CA/CeNPs/Pt1 and (e) CA/CeNPs/Pt5, respectively.

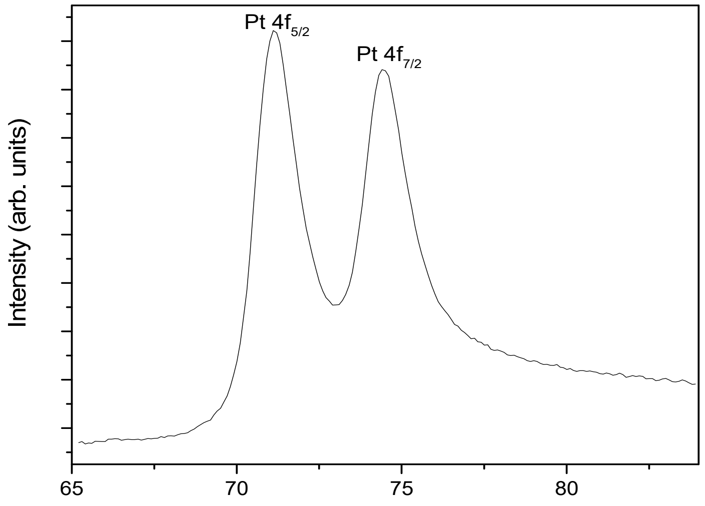

Figure S12. XPS spectra of Platinum rod (99.999% purity) used for SCBD.

The XPS spectra of the pristine rod do not exhibit any signals, which can be referred to the presence of PtO_x species.

Raman Analysis

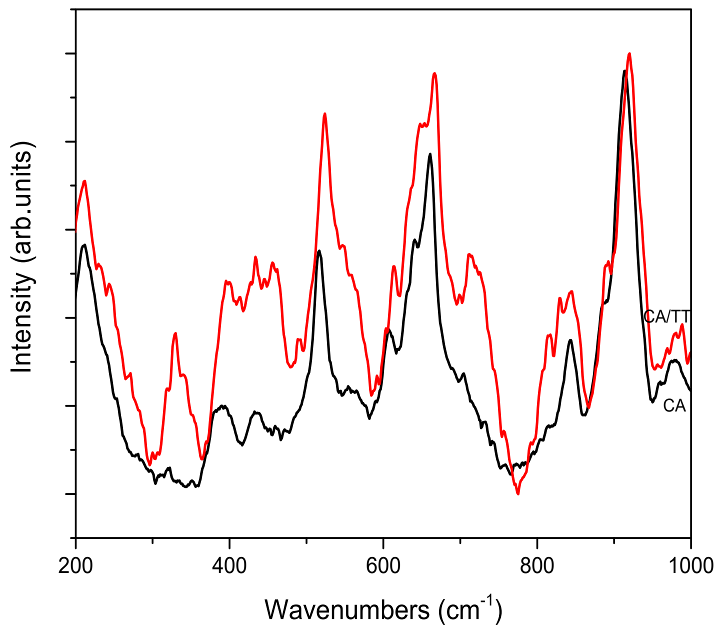

Figure S13. Micro Raman spectra of bare CA (black line) and CA_TT (red line) after the thermal treatment of the polymer at 150°C.

The Raman analysis on the Cellulose Acetate (CA) mats showed different features. The thermal treatment of the membrane induced the formation of polymeric crystalline domains and slightly changed the intensity and the position of the bands. In the table are reported the main vibration signals of CA before and after the thermal treatment [2].

Table S4. Principal vibrational band of Cellulose Acetate matrix

	**CA (neat)**	**CA (therm treat)**
Groups		
-C-O-H-	920 cm^{-1}	917 cm^{-1}
-CCC-	842 cm^{-1}	840 cm^{-1}
$-CH_3CO$	660 cm^{-1}	667 cm^{-1}
-COC-	518 cm^{-1}	522 cm^{-1}
-CO-	Not detected	329 cm^{-1}

Kubelka-Munk approximation

The diffuse-reflectance spectra of the white standard (MgO) and of the samples were collected (Figure S7a), and the R (Reflectance, %) (Eq. S3) and the Kubelka–Munk function *(F)* (Eq. S4) were calculated.

$$R = {}^{Rsample}/_{Rstandard} \quad \text{(eq.S3)}$$

$$F(R) = (1 - R)^{\frac{2}{2R}} = \alpha/S \quad \text{(eq.S4)}$$

F(R) is proportional to the absorption coefficient (α), if it is assumed that the scattering coefficient (S) is approximately constant [3].

The absorption coefficient of semiconductors in the region near the absorption edge is given by Eq. S5, where $h\nu$ is the energy of the incident light and η depends on the type of the optical transition. The η value can be 1/2, 3/2, 2, or 3 for direct-allowed, direct-forbidden, indirect-allowed, and indirect forbidden transitions, respectively.

$$\alpha \propto (h\nu - E_g)^{\eta}/(h\nu) \quad \text{(eq.S5)}$$

Therefore, the absorption edge (band gap energy Eg) can be determined by plotting $(F(R)h\nu)^{1/\eta}$ as a function of $h\nu$. After a linear regression of the obtained plot, the x-intercept reveals the energy of the band gap. We considered a direct-allowed transition, and therefore the $(F(R)h\nu)^2$ versus $h\nu$ was plotted.

Photocatalytic degradation experiments

All the prepared fiber mats and the MB dye solutions were left in contact for 12 hours in the dark inside a quartz-cuvette, (see Experimental Section for further information). The cuvette was irradiated with Visible light (400-600 nm) for 270 min.

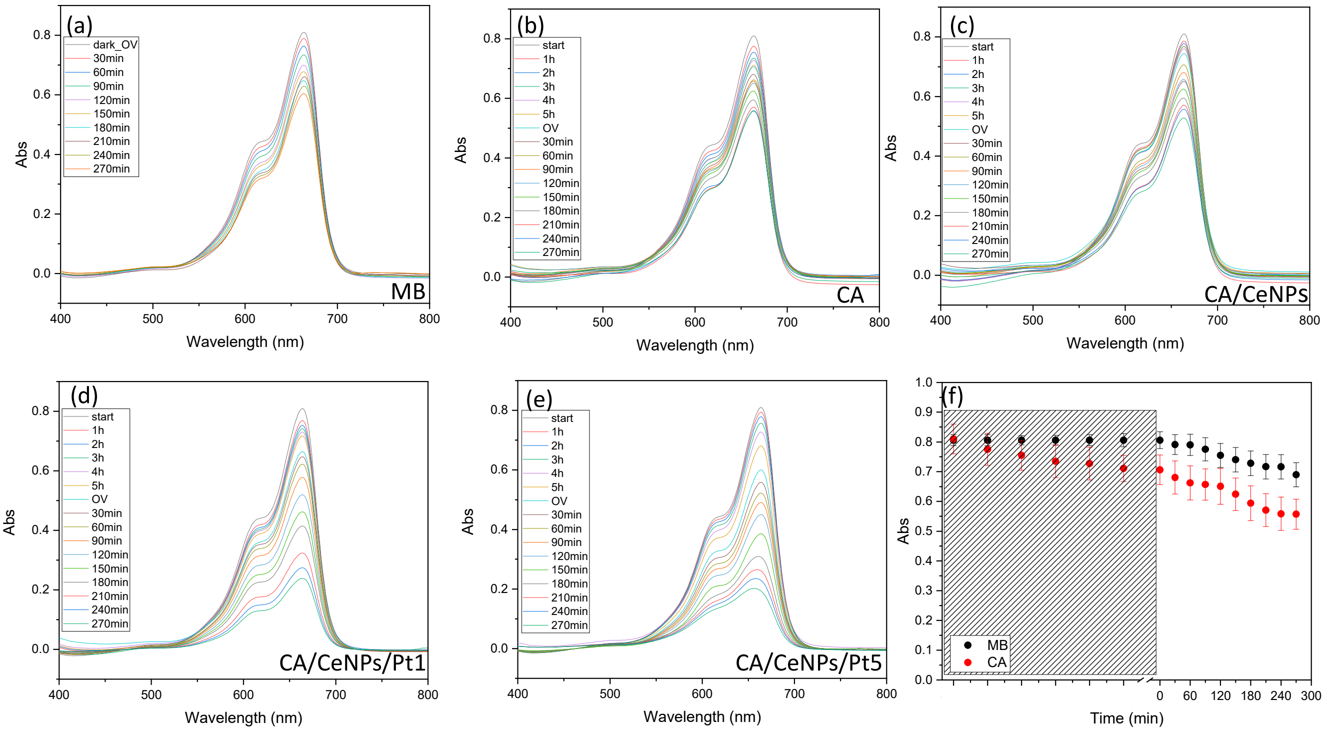

Figure S14. UV-Vis absorption spectra under day-light irradiation, with contact time ranging up 12 hours in dark (the evolution of the absorbance is monitored at the first 5 hours)and subsequent Vis irradiation for 270 minutes .(a) only MB dye (b) MB in presence of neat CA fiber mats, (c) MB in presence of CA/CeNPs, (d) CA/CeNPs/Pt1 and CA/CeNPs/Pt5, respectively. (f) The rate of self-degradation of MB (black dots) and by the presence of CA mats (red dots) is presented.

In the short-time contact experiment, the MB dye was left in contact with the mats for 1 hour, inside a quartz cuvette, and then it was irradiated with Vis lamp for 270 min.

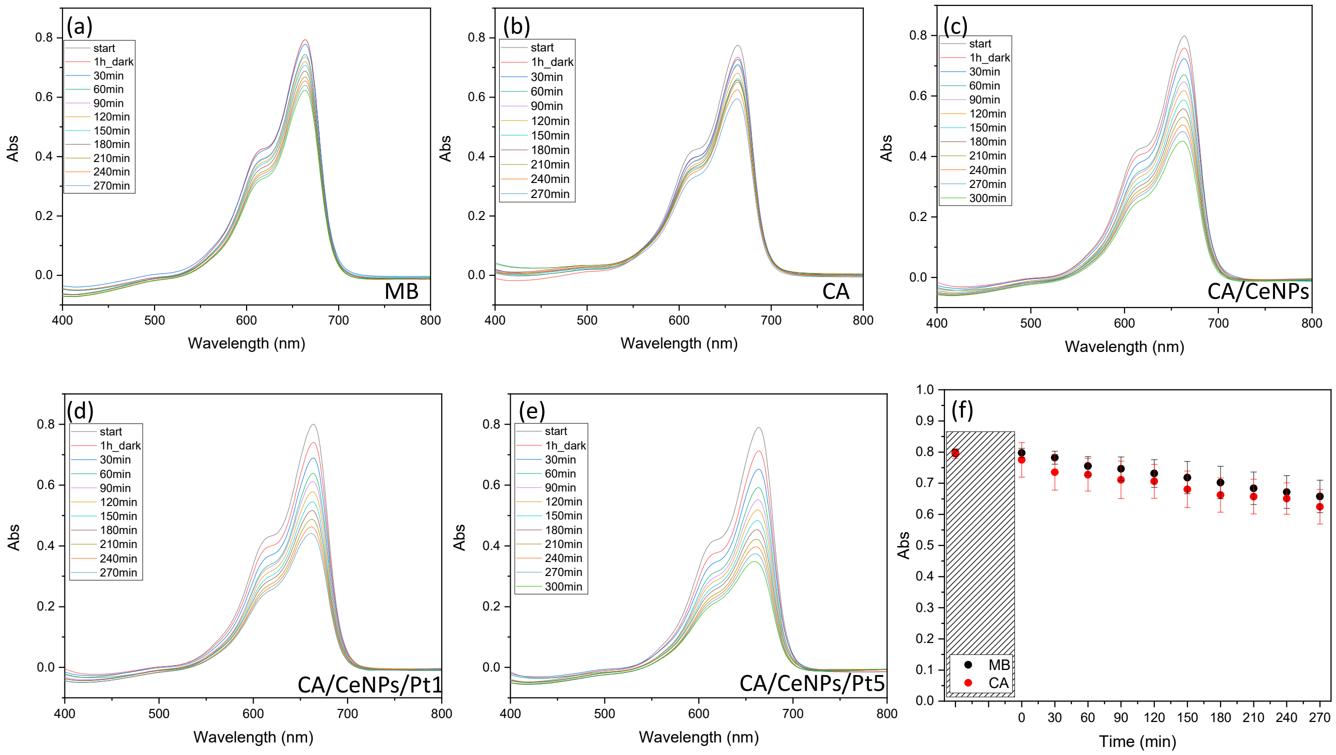

Figure S15. UV-Vis absorption spectra under day-light irradiation, with contact time ranging up 1 hour in dark, and subsequent Vis irradiation for 270 minutes .(a) only MB dye (b) MB in presence of neat CA fiber mats, (c) MB in presence of CA/CeNPs, (d) CA/CeNPs/Pt1 and CA/CeNPs/Pt5, respectively. (f) The rate of self-degradation of the dye (black dots) and by the presence of CA mats (red dots) is presented.

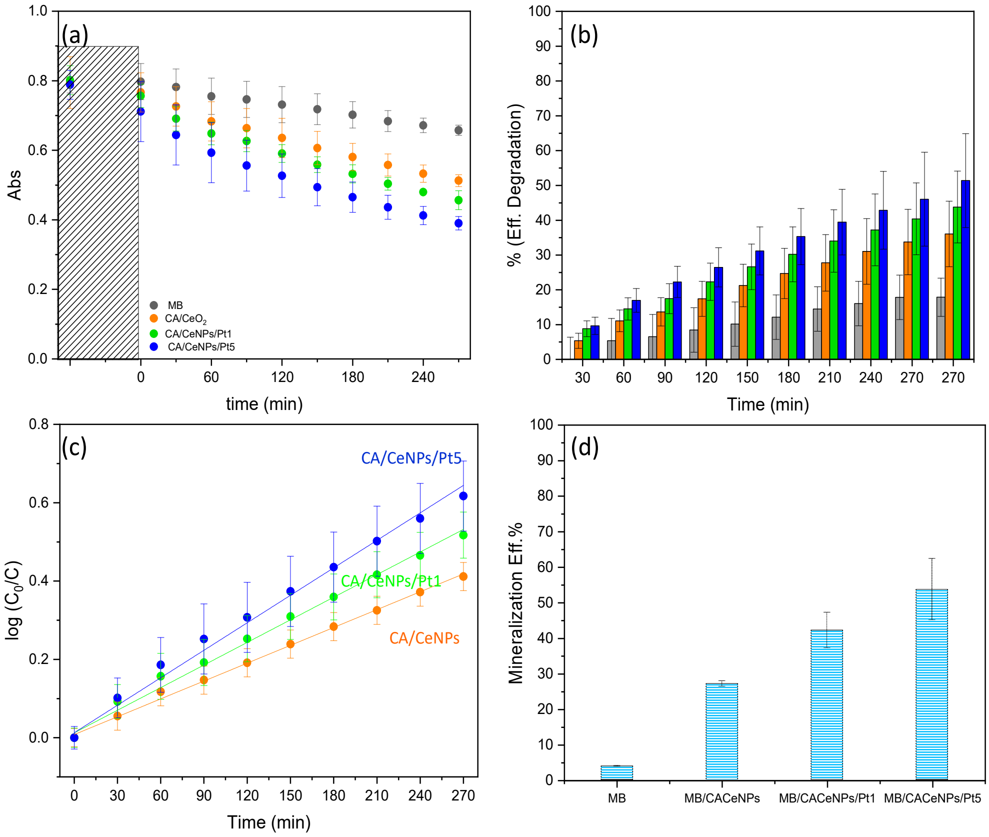

Figure S16. (a) Time dependence of Abs vs. time (b) % of photodegradation and (c) Pseudo first-order kinetic of MB for 1h of time contact with CA/CeNPs/Pt1 (blue dots) and CA/CeNPs/Pt5 (green dots) and (d) percentage (%) of total organic carbon reduction during the irradiation of MB polluted solution after photodegradation process under daylight irradiation.

Table S5. Rate constant of photodegradation process under visible irradiation of all fiber mats at different time contact with MB dye.

**Time contact in dark**	**CA/CeNPs**	**CA/CeNPs/Pt1**	**CA/CeNPs/Pt5**
	k (min^{-1})	*k* (min^{-1})	*k* (min^{-1})
Overnight	$1.3\ 10^{-3}$	$0.7\ 10^{-3}$	$2.4\ 10^{-3}$
		R^2=0.82	R^2=0.99
		$4.7\ 10^{-3}$	$5.9\ 10^{-3}$
R^2	0.99	0.98	0.99
1 hours	$1.6 \cdot 10^{-3}$	$1.9 \cdot 10^{-3}$	$2.4 \cdot 10^{-3}$
R^2	0.99	0.99	0.99

Emission Spectra

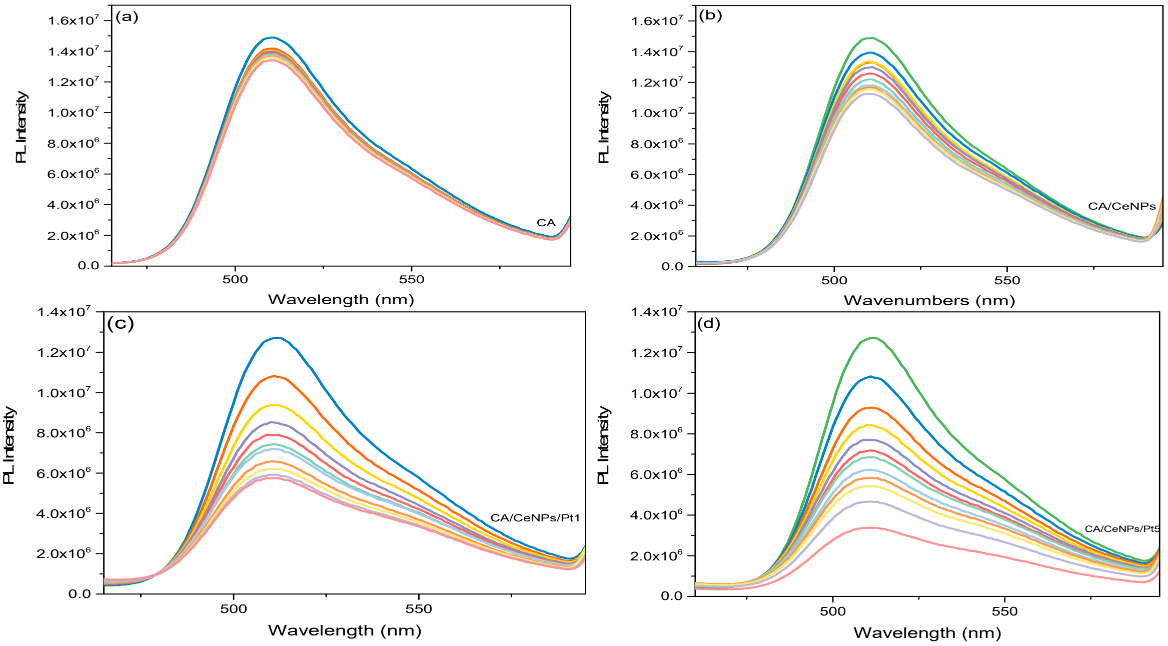

Figure S17. Time-dependent photoluminescence spectra of 8 μM of the aqueous solution of fluorescein sodium (interval time between each measurement: 10 min) in presence of CA/CeNPs, (b) CA/CeNPs/Pt1 and (c) CA/CeNPs/Pt5.

References

1. Mitchell, D.R.G. DiffTools: Electron Diffraction Software Tools for DigitalMicrograph™. *Microscopy Research and Technique* **2008**, *71*, 588–593, doi:https://doi.org/10.1002/jemt.20591.
2. Zhang, K.; Feldner, A.; Fischer, S. FT Raman Spectroscopic Investigation of Cellulose Acetate. *Cellulose* **2011**, *18*, 995–1003, doi:10.1007/s10570-011-9545-8.
3. López, R.; Gómez, R. Band-Gap Energy Estimation from Diffuse Reflectance Measurements on Sol–Gel and Commercial TiO2: A Comparative Study. *J Sol-Gel Sci Technol* **2012**, *61*, 1–7, doi:10.1007/s10971-011-2582-9.
